# Supplementary material for: Deciphering high-order structures in spatial transcriptomes with graph-guided Tucker decomposition
Source: Bioinformatics. 2024 Jun 28;40(Suppl 1):i529–38. doi: 10.1093/bioinformatics/btae245 (PMC11256919; doi:10.1093/bioinformatics/btae245)
Supplement: btae245_Supplementary_Data [file btae245_supplementary_data.zip › btae245_Supplementary_Data/Kuang.108.marked.pdf]

Journal Title Here, 2022, pp. 1–9

doi: DOI HERE
Advance Access Publication Date: Day Month Year
Paper

PAPER

Deciphering High-order Structures in Spatial Transcriptomes with Graph-guided Tucker Decomposition

Charles Broadbent,[1] Tianci Song[1] and Rui Kuang[1,*]

[1]Department of Computer Science and Engineering, University of Minnesota Twin Cities, Minneapolis, 55414, MN, USA
[*]Corresponding author: kuang@umn.edu

Abstract

Spatial transcripome (ST) profiling can reveal cells' structural organizations and functional roles in tissues. However, deciphering the spatial context of gene expressions in ST data is a challenge—the high-order structure hiding in whole transcriptome space over 2D/3D spatial coordinates requires modeling and detection of interpretable high-order elements and components for further functional analysis and interpretation. This paper presents a new method GraphTucker—graph-regularized Tucker tensor decomposition for learning high-order factorization in ST data. GraphTucker is based on a non-negative Tucker decomposition algorithm regularized by a high-order graph that captures spatial relation among spots and functional relation among genes. In the experiments on several Visium and Stereo-seq datasets, the novelty and advantage of modeling multi-way multilinear relationships among the components in Tucker decomposition are demonstrated as opposed to the Canonical Polyadic Decomposition (CPD) and conventional matrix factorization models by evaluation of detecting spatial components of gene modules, clustering spatial coefficients for tissue segmentation and imputing complete spatial transcriptomes. The results of visualization show strong evidence that GraphTucker detect more interpretable spatial components in the context of the spatial domains in the tissues. Availability: `https://github.com/kuanglab/GraphTucker`.

Key words: Spatial transcriptomics, tensor decomposition, high-order analysis

Introduction

In high-throughput spatial transcriptomics (ST) RNA sequencing, spatial gene expressions are profiled from a contiguous population of cells from a tissue. Many ST technologies such as those based on in situ capturing (ISC) allow transcriptome-wide capturing, making it useful for analyzing spatial gene expression patterns with a high coverage of the whole transcriptome [1, 2]. One major complication to ISC-based technologies is that many RNA molecules are failed to be captured due to limitations in the capturing technology, resulting in much sparser gene expressions than what is actually present in the cells. Additionally, identifying spatial patterns can be difficult due to the high dimensionality and high-order organization of the data with complex relations among genes in 2D/3D spatial coordinates. Methods for deciphering these high-order structures and complex relations are therefore important for performing functional analyses of ST data.

Linear matrix factorization models have been successfully shown to offer thorough functional interpretation of ST data [3]. Nonnegative spatial factorization (NSF) is a recently developed probabilistic spatial-aware nonnegative matrix factorization (NMF) model that has been demonstrated to identify important spatial components across ST datasets [4]. NSF and NMF-related models are limited however in their usage of matrix representation of the data, as they collapse the spatial components into a single joint-dimension without capturing higher-order relationships.

Tensor decomposition models do not suffer from this issue as they allow for separate representations of spatial coordinates and genes for capturing higher-order structures. FIST is one such tensor model that finds a linear tensor decomposition of a ST data tensor graph-regularized by a protein-protein interaction network and spatial graphs along x and y dimensions [5]. One drawback of FIST is its use of the canonical polyadic decomposition (CPD) which restricts component interactions across the modes to be a one-to-one match. This restriction not only over-simplifies the high-order structure with a strong assumption across the modes but also significantly reduce the interpretability of the detected components, such that the interpretations of the CPD model learned on ST data rely on the imputation of the whole tensor for other analyses [5, 6].

In this work, we present GraphTucker, a graph-regularized Tucker decomposition model which offers a novel approach for higher-order interpretation of ST data allowing multi-way relations among the components across the spatial modes and the gene mode. As outlined in comparison with other factorization models in Figure 1, GraphTucker finds a Tucker tensor decomposition with a nonnegative factorization algorithm which

© The Author 2022. Published by Oxford University Press. All rights reserved. For permissions, please e-mail: journals.permissions@oup.com

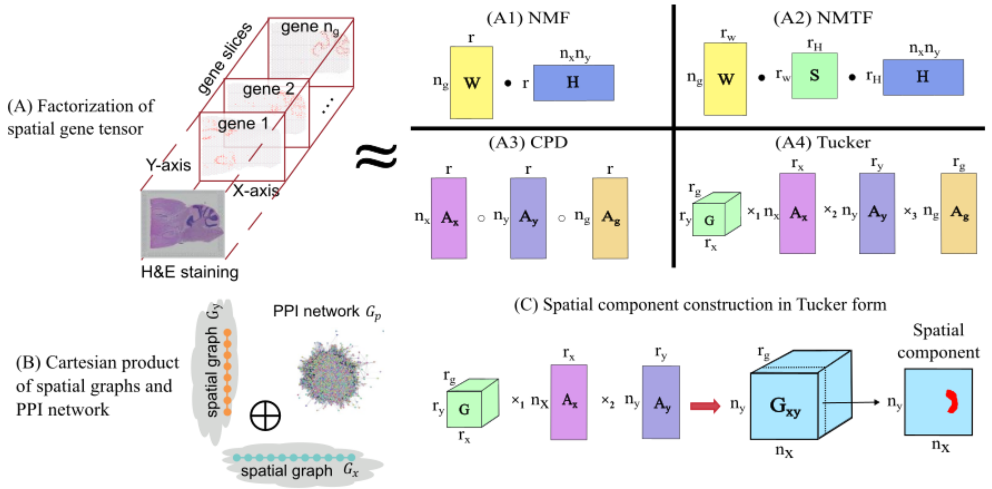

Fig. 1. Overview of factorization of ST data with graph regularization. (A) Factorization of spatial gene expression tensor using four different methods. These include two matrix factorizations, (A1) Nonnegative matrix factorization (NMF), (A2) Nonnegative matrix tri-factorization (NMTF), and two tensor decomposition methods, (A3) Canonical polyadic decomposition (CPD), and (A4) Tucker decomposition. (B) The Cartesian product is computed between spatial graphs in the x and y coordinates, and a PPI network. This product graph is used to apply graph regularization to the Tucker decomposition optimization. (C) Spatial components are constructed using the core tensor and the x and y factor matrices. $\cdot$ denotes matrix multiplication, $\circ$ denotes component-wise outer product, and $\times_n$ denotes the n-mode tensor product.

simultaneously reconstructs the tensor and exploits relationships among the spatial coordinates and genes. The component matrices and the core tensor in the Tucker decomposition can be aggregated to derive interpretable spatial components that capture important higher-order spatial gene expression activity maps as well as reconstructing the ST data. In the experiments, we demonstrate GraphTucker is an effective and scalable method for application to 10x Genomics Visium data and high-resolution Stereo-seq data.

Note that deep-learning models for resolving spatial transcriptomes have been growing in popularity, with methods such as STAGATE, Tangram, ST-Net, Hist2ST, stPlus, and GNTD being used with varying types of neural-network models [6, 7, 8, 9, 10, 11]. Although these methods have been shown to perform well in their respective contexts, none of the deep neural-network models provide interpretation of the structures in the original ST data such as spatial components or spatial context for gene modules.

Methods

In this section, we first introduce the GraphTucker model and its non-negative factorization algorithm, and then the derivation of the spatial components from the Tucker factorization. Note that while the formulations are only shown for 3-way tensors for conciseness, they can be directly generalized to higher orders.

Preliminaries

GraphTucker consists of two main components, a Tucker tensor decomposition (Fig. 1A) and Cartesian product graph regularization (Fig. 1B), which are reviewed below. A more complete background review is also given in Section S1 in Supplementary Materials for readers' interest.

Tucker decomposition

Given a third order tensor $\boldsymbol{X} \in \mathbb{R}^{I \times J \times K}$, the **Tucker decomposition** seeks to approximate $\boldsymbol{X}$ in the following form:

$$\boldsymbol{X} \approx [\![\boldsymbol{G}; \boldsymbol{A}, \boldsymbol{B}, \boldsymbol{C}]\!] \equiv \sum_{i=1}^{R_a} \sum_{j=1}^{R_b} \sum_{k=1}^{R_c} g_{ijk} \, \boldsymbol{a}_i \circ \boldsymbol{b}_j \circ \boldsymbol{c}_k,$$

where R_a, R_b, and R_c are the number of components/columns in the factor matrices $\boldsymbol{A}, \boldsymbol{B}$ and $\boldsymbol{C}$, respectively, such that $\boldsymbol{A} \in \mathbb{R}^{I \times R_a}$, $\boldsymbol{B} \in \mathbb{R}^{J \times R_b}$, and $\boldsymbol{C} \in \mathbb{R}^{K \times R_c}$, and $\boldsymbol{G} \in \mathbb{R}^{R_a \times R_b \times R_c}$. The Tucker decomposition allows for factor matrices to have different number of components, and we notate this by saying $\boldsymbol{X}$ has rank $= (R_a, R_b, R_c)$. $\boldsymbol{G}$ is known as the **core tensor**, and contains values indicating the multi-way interactions among the components from each of the factor matrices.

Cartesian Graph regularization

Cartesian product graph regularization has been previously shown to improve spatial gene expression imputation [5]. In this setting of ST data factorization, the Cartesian product graph $\mathcal{G}(x, y, g)$ is calculated as the Cartesian product of three graphs: a protein-protein interaction (PPI) network G_g, and two unweighted, undirected chain graphs G_x and G_y which correspond to the x and y components of the grid. To compute the Laplacian $\mathcal{L}(x, y, g)$ of the Cartesian product graph we take the Kronecker sum of the Laplacians of each of the three graphs i.e. $\mathcal{L}(x, y, g) = L_x \oplus L_y \oplus L_g$, where each Laplacian matrix L_i of a graph G_i is calculated as $L_i = D_i - W_i$ with D_i and W_i being the degree and adjacency matrices of G_i. This Laplacian of the product graph can impose a high-order regularization on a Tucker decomposition $\hat{\boldsymbol{X}}$ as

$$\mathrm{vec}(\hat{\boldsymbol{X}})^T \mathcal{L}(x, y, p) \mathrm{vec}(\hat{\boldsymbol{X}}),$$

where $\mathrm{vec}(\mathbf{X})$ denotes vectorization of a tensor $\mathbf{X}$. This regularization term helps enforce two assumptions about how the data is laid out in the tensor: co-expressed (or the same) genes should share similar expression levels at adjacent spots.

GraphTucker: Graph-regularized Tucker Decomposition

Given a ST tensor, $\boldsymbol{T} \in \mathbb{R}_+^{n_x \times n_y \times n_g}$ where n_x, n_y and n_g denote the number of spots along x-coordinate and y-coordinate and the number of genes respectively, GraphTucker solves the following

minimization problem:

$$\min_{\{\boldsymbol{G},\mathbf{A}_y,\mathbf{A}_x,\mathbf{A}_g\}} \frac{1}{2}||\boldsymbol{M} \odot (\boldsymbol{T} - \hat{\boldsymbol{T}})||_F^2 + \frac{\lambda}{2}\text{vec}(\hat{\boldsymbol{T}})^T \mathbf{L}_c \text{vec}(\hat{\boldsymbol{T}}) \quad \text{s.t. } \boldsymbol{G}, \mathbf{A}_y, \mathbf{A}_x, \mathbf{A}_g \geq 0, \tag{1}$$

where $\hat{\boldsymbol{T}} = [\![\boldsymbol{G}; \mathbf{A}_y, \mathbf{A}_x, \mathbf{A}_g]\!] \in \mathbb{R}_+^{n_y \times n_x \times n_g}$ is the rank=(r_y, r_x, r_g). Tucker decomposition of the original data tensor $\boldsymbol{T}$, $\mathbf{A}_y \in \mathbb{R}_+^{n_y \times r_y}, \mathbf{A}_x \in \mathbb{R}_+^{n_x \times r_x}$ are the x and y factor matrices, $\mathbf{A}_g \in \mathbb{R}_+^{n_g \times r_g}$ is the gene component factor matrix, $\boldsymbol{G} \in \mathbb{R}_+^{r_y \times r_x \times r_g}$ is the core tensor as defined in the Tucker model, $\boldsymbol{M} \in \mathbb{R}_{[0,1]}^{n_y \times n_x \times n_g}$ is a binary mask tensor for selecting observed entries in both spatial gene expression tensors, and $\mathbf{L}_c = \mathcal{L}(g, y, x) = \mathbf{L}_g \oplus \mathbf{L}_y \oplus \mathbf{L}_x$ is the Laplacian matrix of the Cartesian product of a PPI graph and each spatial chain graphs in the x and y modes.

We propose an iterative algorithm GraphTucker in Algorithm 1 that updates the factor matrices and core tensor in succession, computing the partial derivatives for one while fixing all others. GraphTucker takes in four main inputs: the incomplete spatial gene expression tensor $\boldsymbol{T}$, a binary mask tensor $\boldsymbol{M}$ for selecting observed entries in $\boldsymbol{T}$, PPI network G_g, and hyperparameter λ for controlling the influence of the graph regularization on the imputation. The output is the Tucker decomposition of $\boldsymbol{T}$ composed of $\mathbf{A}_y, \mathbf{A}_x, \mathbf{A}_g$, and $\boldsymbol{G}$.

Algorithm 1 GraphTucker

Inputs:
Spatial gene expression tensor $\boldsymbol{T} \in \mathbb{R}_+^{n_y \times n_x \times n_g}$, binary mask tensor $\boldsymbol{M} \in \mathbb{R}_{\{0,1\}}^{n_y \times n_x \times n_g}$ for selecting observed entries, PPI network G_g, hyperparameter λ.

Initialize:
Randomly initialize $\mathbf{A}_y \in \mathbb{R}_+^{n_y \times r_y}$, $\mathbf{A}_x \in \mathbb{R}_+^{n_x \times r_x}$, $\mathbf{A}_g \in \mathbb{R}_+^{n_g \times r_g}$ and $\boldsymbol{G} \in \mathbb{R}_+^{n_y \times n_x \times n_g}$. Construct spatial chain graphs G_x and G_y.

while not converged **do**

$$[\mathbf{A}_y]_{i,j} \longleftarrow [\mathbf{A}_y]_{i,j} \left(\frac{\left[\frac{\partial \mathcal{F}_1}{\partial \mathbf{A}_y}\right]^-_{i,j} + \lambda \left[\frac{\partial \mathcal{F}_2}{\partial \mathbf{A}_y}\right]^-_{i,j}}{\left[\frac{\partial \mathcal{F}_1}{\partial \mathbf{A}_y}\right]^+_{i,j} + \lambda \left[\frac{\partial \mathcal{F}_2}{\partial \mathbf{A}_y}\right]^+_{i,j}} \right) \text{ (Eq. S2.(20))}$$

$$[\mathbf{A}_x]_{i,j} \longleftarrow [\mathbf{A}_x]_{i,j} \left(\frac{\left[\frac{\partial \mathcal{F}_1}{\partial \mathbf{A}_x}\right]^-_{i,j} + \lambda \left[\frac{\partial \mathcal{F}_2}{\partial \mathbf{A}_x}\right]^-_{i,j}}{\left[\frac{\partial \mathcal{F}_1}{\partial \mathbf{A}_x}\right]^+_{i,j} + \lambda \left[\frac{\partial \mathcal{F}_2}{\partial \mathbf{A}_x}\right]^+_{i,j}} \right) \text{ (Eq. S2.(19))}$$

$$[\mathbf{A}_g]_{i,j} \longleftarrow [\mathbf{A}_g]_{i,j} \left(\frac{\left[\frac{\partial \mathcal{F}_1}{\partial \mathbf{A}_g}\right]^-_{i,j} + \lambda \left[\frac{\partial \mathcal{F}_2}{\partial \mathbf{A}_g}\right]^-_{i,j}}{\left[\frac{\partial \mathcal{F}_1}{\partial \mathbf{A}_g}\right]^+_{i,j} + \lambda \left[\frac{\partial \mathcal{F}_2}{\partial \mathbf{A}_g}\right]^+_{i,j}} \right) \text{ (Eq. S2.(19))}$$

$$[\mathbf{vec}(\mathbf{G}_{(g)})]_i \leftarrow [\mathbf{vec}(\mathbf{G}_{(g)})]_i \left(\frac{\left[\frac{\partial \mathcal{F}_1}{\partial \mathbf{vec}(\mathbf{G}_{(g)})}\right]^-_{i} + \lambda \left[\frac{\partial \mathcal{F}_2}{\partial \mathbf{vec}(\mathbf{G}_{(g)})}\right]^-_{i}}{\left[\frac{\partial \mathcal{F}_1}{\partial \mathbf{vec}(\mathbf{G}_{(g)})}\right]^+_{i} + \lambda \left[\frac{\partial \mathcal{F}_2}{\partial \mathbf{vec}(\mathbf{G}_{(g)})}\right]^+_{i}} \right) \text{ (Eq. S1. (28))}$$

$$[\![\boldsymbol{G}; \mathbf{A}_y, \mathbf{A}_x, \mathbf{A}_g]\!] \leftarrow [\![\boldsymbol{G} \times_1 \mathbf{U} \times_2 \mathbf{V} \times_3 \mathbf{W}; \mathbf{A}_y\mathbf{U}^{-1}, \mathbf{A}_x\mathbf{V}^{-1}, \mathbf{A}_g\mathbf{W}^{-1}]\!] \text{ // normalization (S3.(29))}$$

end while

Return: $\mathbf{A}_y, \mathbf{A}_x, \mathbf{A}_g, \boldsymbol{G}$

GraphTucker minimizes the optimization problem in Eq. (1) using a multiplicative updating (MU) approach to maintain nonnegativity. GraphTucker starts by first computing the gradient of Eq. (1) w.r.t. $\mathbf{A}_y$ while fixing $\mathbf{A}_x, \mathbf{A}_g$, and $\boldsymbol{G}$. The MU rule is derived based on the positive and negative components of the gradient which are then used to update $\mathbf{A}_y$. This updating process continues similarly by finding MU rules for $\mathbf{A}_x, \mathbf{A}_g$, and then $\boldsymbol{G}$, in that order. Complete derivations of MU rules for the factor matrices and core tensor are provided in Equations S2.(12)-(28) in the Supplementary Materials. GraphTucker runs either until convergence or a maximum number of iterations has been reached, which we set to 5000. Convergence is determined if the residuals between iterations is less than 10^{-4}.

To improve interpretability and convergence, a normalization step is performed at the end of every iteration: the sums of each column in each factor matrix are multiplied in the corresponding entries in the core tensor to represent the magnitude of each multi-way interaction and then each column in each factor matrix is normalized to sum to 1 (Eq. S3.(29)).

Spatial component construction

Using the resulting regularized Tucker decomposition found by GraphTucker, we define the *spatial components* tensor $\boldsymbol{G}_{xy}$ as shown in Fig. 1C:

$$\boldsymbol{G}_{xy} = \boldsymbol{G} \times_1 \mathbf{A}_x \times_2 \mathbf{A}_y, \tag{2}$$

where $\boldsymbol{G}_{xy}$ contains r_g spatial components which each correspond to a gene component in $\mathbf{A}_g$, meaning each spatial component is composed of all combinations of interactions between a particular gene component $[\mathbf{A}_g]_{:,i}$ and all components of $\mathbf{A}_x$ and $\mathbf{A}_y$. Spatial components and their coefficients therefore indicate activities of each gene component at the locations in a tissue. To analyze strong multiway interactions, as indicated by large values in the core tensor, a sparse core tensor can also be constructed by keeping the largest $n\%$ of entries and setting all other entries to zero. To only use the strong interactions, the spatial components can then be constructed by replacing $\boldsymbol{G}$ with $\boldsymbol{G}^n$ in Eq. 2, where n indicates the top $n\%$ of entries kept in the core tensor.

Experiments

In the experiments, we measure GraphTucker's performance by evaluating its ability to estimate missing or holdout gene expression entries and capture known tissue structures by the spatial components and the spatial coefficients of the gene modules on several human and mouse 10x Genomics Visium and Stereo-seq datasets. Multiple linear factorization models and a CPD model are compared against GraphTucker as baselines.

Data preparation

The datasets used in this study are listed in Table 1 with specifications of the size, density, annotation, platform and source of each dataset. The MBSA, MBSP and BRCA1 datasets were downloaded from 10x Genomic website [12]. The BRCA1 data was manually annotated into 20 tissue regions [13]. The DLPFC Human Brain dataset was generated and annotated in the spatialLIBD project [14, 15, 16]. Two Stereo-seq mouse embryo datasets were dowloaded from **MOSTA** with anatomical regions annotated based on cell segmentation with nucleic acid staining image followed by unsupervised spatial clustering and analysis of known spatially variable marker genes [17].

Given the hexagonal grid structure of the Visium protocol, the datasets were adjusted by shifting odd-numbered rows by half a spot to give a square grid. All gene expressions from a tissue are then arranged into a three-way tensor $\boldsymbol{T} \in \mathbb{R}^{n_g \times n_y \times n_x}$. Extremely lowly expressed entries (less than 3 UMI counts) are set to 0. Low density genes with total UMI counts less than 4 were also removed for better computational efficiency in the datasets used in the imputation experiment and the larger Stereo-seq datasets used in the case study. Rows and columns of all empty spots were also cropped out and removed. Each entry in the tensor was then log-transformed as $T_{g,x,y} \leftarrow \log(T_{g,x,y} + 1)$. The size of the processed data is given in Table 1.

Table 1. Summary of datasets.

Dataset	Size (n_x, n_y, n_g)	Density	Annotation	Platform	Source
Mouse Brain Section 1, Sagittal-Anterior (MBSA)	(66, 59, 11327)	0.1239	NA	10x Visium	[12]
Mouse Brain Section 1, Sagittal-Posterior (MBSP)	(67, 62, 11285)	0.0987	NA	10x Visium	[12]
Human Breast Cancer, Block A Section 1 (BRCA1)	(60, 77, 12125)	0.1204	20 regions	10x Visium	[12]
DLPFC Human Brain 151673	(59, 76, 17891)	0.0134	7 layers	10x Visium	[14, 15, 16]
Mouse Embryo E.9.5 E1S1 (Day 9.5)	(80, 107, 9687)	0.0703	11 organs	Stereo-seq	[17]
Mouse Embryo E.11.5 E1S1 (Day 11.5)	(187, 264, 10023)	0.0392	18 organs	Stereo-seq	[17]

Version 4.4.226 *Homo sapien* and *Mus musculus* PPI networks were acquired from BioGRID and used for human and mouse datasets, respectively [18]. PPI networks were overlapped with each dataset based on intersection between the genes remaining after preprocessing and the genes connected in the network.

Compared methods

We used three methods based on the factorizations shown in Fig. 1A as well as NSFH as baseline comparisons, focusing on linear methods that are able to extract spatial components in spatial transcriptomics data so that they can be used for direct quantitative and visual comparison to GraphTucker.

- **Nonnegative Matrix Factorization (NMF)** factorizes a matrix Y into two matrices W, H such that $Y = WH$ (panel A1 in Fig. 1A). The number of columns in W and rows in H is denoted as the *rank*, which can be tuned according to the needed approximation accuracy. We apply NMF by flattening the spatial gene expression tensor $\boldsymbol{T} \in \mathbb{R}_+^{n_x \times n_y \times n_g}$ into a matrix $Y \in \mathbb{R}_+^{n_g \times n_x n_y}$, and factorize it into a gene component matrix $W \in \mathbb{R}_+^{n_g \times r}$ and spatial component coefficient matrix $H \in \mathbb{R}_+^{r \times n_x n_y}$, where r is the rank. We use the MATLAB function `nnmf()`, which is an implementation of the algorithm in Berry et al. [19], for testing NMF.
- **Regularized Nonnegative Matrix Tri-Factorization (RNMTF)** is a regularized Nonnegative Matrix Tri-Factorization (NMTF), which is an altered NMF that allows for multiway interactions between gene and spatial components through the addition of a core matrix S, such that $Y = WSH$ (panel A2 in Fig. 1A). This also allows W and H to have their own ranks r_W and r_H, such that $S \in \mathbb{R}_+^{r_W \times r_H}$. We adopted RNMTF [20] to include regularization to NMTF by introducing graph regularization into the optimization, where W can be seperatedly regularized by a PPI network and H by a x/y spatial graph. These regularizations have corresponding hyperparameters γ and ρ, which we tuned to minimize approximation error.
- **Nonnegative spatial factorization (NSF)** is a new method that extends NMF to be both probabilistic and spatially-aware by modeling Gaussian processes across spatial data, and was demonstrated for recovering spatial factors in ST data [4]. NSF factorizes a matrix $Y = FW$, where F is the spatial factors matrix with L components that models a Gaussian Process prior over the factors, and W is the loadings matrix. A hybrid version (NSFH) was also created that allows the separation of spatial factors into nonspatial and spatial ones. We only use the spatial factors as we do not expect nonspatial factors to provide useful components for spatial component analysis. We followed the same pre and post-processing experimental setup as used in their experiments to obtain the spatial factors (components) using the top 2000 genes as determined by Poisson deviance. We also run NSFH with the number of inducing points (IPs) equal to the number of observations (spots) in each tested dataset for the full resolution.
- **Fast Imputation of Spatially-resolved transcriptomes by graph-regularized Tensor completion (FIST)** is a recently developed method that uses the canonical polyadic decomposition (CPD) for finding a regularized tensor decomposition of spatial transcriptomics data (panel A3 in Fig. 1A) [5]. FIST differs from GraphTucker mainly in its ability to find multilinear relationships between its components. FIST spatial components are computed by taking the outer product between the rank-r pairs of components in its x and y factor matrices.

A note is that running FIST with $\lambda = 0$ is equivalent to computing the nonnegative CPD of the given tensor without regularization. Similarly, running GraphTucker with $\lambda = 0$ is equivalent to computing the nonnegative Tucker decomposition without regularization. We tested both settings to demonstrate the importance of including graph regularization.

Imputation by spotwise cross-validation

We evaluated the generalization of GraphTucker, RNMTF, and FIST by running 5-fold cross validation across three Visium datasets: MBSA, MBSP, and BRCA1. Cross-validation was performed spotwise where 20% of spots are held out for testing by setting their entries equal to 0, with the remaining 80% of spots used for training. Spots which contain zero gene expression across all genes were not selected for testing. In FIST and GraphTucker, test spots and all-zero spots were excluded from training by using the mask tensor. NMF was not compared against in this experiment since the removal of whole spots completely prevents any imputation in its factorization. NSFH was also not compared since its setting was only on 2000 genes in all the experiments in the original study [4].

Spatial component analysis

We analyzed GraphTucker's spatial components by comparing them against ground-truth annotated regions in two Visium datasets, BRCA1 Human Breaset Cancer dataset and DLPFC Human Brain dataset, expecting good spatial components can reveal detailed domain structures for biological interpretation of spatial augmentations corresponding to the annotations. First, after running each method on both datasets, we matched each annotated region to a corresponding spatial component by calculating the Area Under the receiver operating Curve (AUC) and Euclidean Distance (ED) pairwise between every region and spatial component. Matches were selected by choosing the pairing with the lowest ED.

Spatial domain detection

In addition, we also performed k-means clustering on each method's spatial components across the spots using $k = 7$ and $k = 20$ to match the number of regions on the BRCA1 and DLPFC datasets, respectively. We then compared the resulting clusterings to the ground truth annotations to measure

how closely each method's spatial components captured the annotated regions.

Evaluation metrics

Three metrics were used to evaluate cross-validation performance: mean absolute error (MAE), mean average percent error (MAPE), and coefficient of determination (R^2) (Eq. S4.(30)). We use MAE and MAPE to measure how accurate each method approximates missing gene expression values, and R^2 is used to measure how imputed expressions correlate with the original expressions across a spot.

AUC was calculated by binarizing the annotated region on the tissue, such that every spot in the region has a value of 1 and 0 everywhere else. AUC was then calculated by thresholding a spatial component using the binary annotation as labels for each spot. Euclidean distance (ED) was calculated by normalizing both the binary annotation and spatial component to length 1, and then computing the Euclidean distance between the two. AUC and ED are used to measure how closely a spatial component corresponds to a region, with an AUC of 1 and ED of 0 indicating a perfect representation of the region.

In the clustering experiments, we computed the Adjusted Rand Index (ARI) between these clusterings and the original clusterings (Eq. S4.(31)). An ARI closer to 1 indicates the spatial component clusterings better match the original clusters.

Parameter tuning

In the imputation experiments, we chose to use rank= 50 for factorization with RNMTF, FIST, and GraphTucker since previous extensive experimentation with decomposition analysis of the datasets showed that 50 components are sufficient for achieving good imputation and using higher ranks do not lead to consistent improvement [6]. GraphTucker was run with rank= (50, 50, 50) for a high resolution in x/y coordinates. FIST and GraphTucker were both tuned with and without graph regularization, and we report the results for $\lambda = 0.1$ as it gave stable performance across all the datasets as previously observed [6, 5]. We also tuned γ and ρ for RNMTF, but observed best performance with $\rho = 0$ and $\gamma = [0.1, 1, 10]$ for MBSA, MBSP1, and BRCA1 respectively.

For the spatial component analyses, each method was run with the number of gene components equal to the number of regions. GraphTucker was run with rank= $(30, 30, 7)$ and rank= $(30, 30, 20)$ on the human brain and breast cancer datasets, respectively, as we observed that 30 x/y components provides a sufficient resolution for detecting the low number of gene components. Specifically, on the human brain dataset, NMF was run with rank=7, RNMTF with rank=(7,50), and NSFH with L=7. Similarly on the breast cancer dataset, NMF was run with rank=20, RNMTF with rank=(20,50), NSFH with L=20. We also tested NMF, RNMTF at other ranks around the number of ground-truth regions. The detected components are not significantly different and thus, we only show the results for rank 7 and 20 on the two datasets, respectively. Since FIST is inflexible in choosing rank in each mode, we tested a larger rank=50 and reported the lower average ED in the comparison. We tuned λ for both GraphTucker and FIST and found $\lambda = 1$ to give the best results for both AUC and ED. RNMTF was run with $\rho = 0, \gamma = 0.1$ the same optimal parameters in the imputation experiments.

In the case study on the two Stereo-seq mouse embryos, we ran GraphTucker with rank= $(64, 64, 64), \lambda = 0.1$ for the higher resolution of the spot grid, and complex annotations of the organs in the mouse embryos, around 50 annotated regions depending the developmental day. We then manually analyzed the resulting spatial components to look for visual matches to any of the annotated regions.

Results

GraphTucker imputes missing spatial gene expressions in Visium data more accurately

We benchmarked GraphTucker against the baseline methods to evaluate its performance in cross-validation experiments across three 10x Genomics Visium datasets including mouse brain sagittal-anterior (MBSA) and sagittal-posterior (MBSP) datasets, and a human breast cancer (BRCA1) dataset. All methods were evaluated by three performance metrics, MAE, MAPE, and R^2 across all datasets as shown in Table 2.

In terms of MAE and MAPE, GraphTucker outperforms both RNMTF and FIST across all three datasets. As expected, GraphTucker and FIST perform better using graph regularization than without, with GraphTucker achieving improved approximation over FIST when using $\lambda = 0.1$. This difference in performance is less pronounced on the BRCA1 dataset, although GraphTucker is still slightly better. RNMTF performs significantly worse than FIST and GraphTucker, which is expected as it was not originally developed for generalization in a spotwise cross-validation context.

GraphTucker similarly outperforms FIST in terms of R^2, demonstrating it can better impute missing values that correlate more closely to missing gene expressions. In datasets with highly sparse expressions across even a small number of genes, a poor generalization for these genes can result in very low and sometimes negative R^2 as seen in RNMTF.

GraphTucker detects spatial components that capture known tissue regions in annotated Visium data

To analyze the GraphTucker's components, we evaluated how closely its spatial components captured known tissue regions in the two annotated Visium datasets from the human brain tissues consisting of seven regions, and the human breast cancer tissue with twenty regions. The results are visualized in Figure 2 and Figure 3 for the two datasets, respectively. We also found that using the top 10% and 5% of core tensor entries in the human brain and breast cancer datasets respectively gave lower ED and and higher AUC on average across matched spatial components as shown in Figure S1 and S2. Despite using such a small amount of entries in the core tensor, they accounted for 0.835 and 0.684 of total interactions respectively for the two datasets.

As shown in Table 3, GraphTucker using the top 10% and 5% of core tensor entries had the best average AUC and ED of its region-spatial component matches on the human brain and BRCA1 datasets. This suggests GraphTucker can sufficiently capture a wide variety of regions in its components, even with a high number of closely packed regions such as in the breast cancer tissue. Furthermore, these results indicate that many of these spatial domains can be captured by a relatively low number of component interactions of high importance, which GraphTucker excels at finding.

In the human brain dataset, GraphTucker achieves the highest AUC and lowest ED in regions L3 and L6, as well as the highest AUC in L1 and L4 and lowest ED in WM, as shown in Figure 2. NSFH has the best performance for the L5 region, and highest AUC for the WM. Lastly, NMF best captures the L2 region. An important note is that GraphTucker's and NSFH's spatial components are noticeably smoother compared

Table 2. Spotwise cross-validation results across three Visium datasets. The best results for each metric are marked in bold.

	MBSA			MBSP			BRCA1		
**Methods**	**MAE**	**MAPE**	**R^2**	**MAE**	**MAPE**	**R^2**	**MAE**	**MAPE**	**R^2**
RNMTF, rank=(50,50)	1.19	0.69	-2.12	1.07	0.64	-1.77	1.08	0.64	-1.67
FIST, rank=50, $\lambda = 0$	0.33	0.21	0.61	0.35	0.22	0.39	0.31	0.20	0.61
FIST, rank=50, $\lambda = 0.1$	0.30	0.19	0.68	0.33	0.21	0.41	**0.30**	0.19	0.68
GraphTucker, rank=(50,50,50), $\lambda = 0$	0.34	0.21	0.56	0.36	0.23	0.32	0.31	0.19	0.61
GraphTucker, rank=(50,50,50), $\lambda = 0.1$	**0.29**	**0.18**	**0.71**	**0.31**	**0.20**	**0.49**	**0.30**	**0.18**	**0.70**

to NMF due to their respective usage of graph regularization and Gaussian Process prior.

In the breast cancer dataset, we report visualizations and metrics for eight of the twenty regions in Figure 3. GraphTucker achieves the highest AUC and lowest ED in six of the shown regions, DCIS-LCIS1, DCIS-LCIS 4, IDC 2, IDC 5, IDC6, and IDC7, as well as the highest AUC in DCIS-LCIS 3. NSFH best captures IDC 4 and FIST achieves the lowest ED in DCIS-LCIS 3. Complete results for all twenty regions are provided in Figure S3. An important note is that some regions are captured best by the same component, which we observed in regions IDC 6 and IDC 7, as both NMF, FIST, and GraphTucker captured these regions in the same spatial component.

On the Human Brain dataset, we obtained a curated layer-specific marker gene list from Maynard et al. [15] and compared the top genes in the matched gene components of GraphTucker, NMF, RNMTF, and FIST. We excluded WM and L1 regions in this analysis since they only have five marker genes each. We found significant overlap in all the other five regions L2-L6. NSFH is not applicable in this analysis as only 25 of the 126 marker genes were present in the 2000 genes pre-selected for training. Figure S3 shows that GraphTucker is able to highly rank layer-specific marker genes in its gene components, and overall finds these genes better than the other methods. On a region-by-region basis, in some cases NMF and FIST have slightly larger overlap (see p-values in Table S2-4). Regardless, these results provide additional evidence that GraphTucker gene components capture the layer-specific expressions by recovering many marker genes known in the literature.

For the six tumor edge regions, we observed only three GraphTucker spatial components to match to these six regions, with four regions being best captured by a single component. Since each component alone was not sufficiently visualizing any one region, we combined all three components using all core tensor entries and averaged them to determine how well the tumor edges were being captured as a whole, which is shown in Figure 4. These averaged components provide a significantly better visualization of the tumor edge regions than when separated. Although the spatial components constructed from the top 5% of core tensor entries can approximate most of the tumor edge regions, using more entries gives a better overall segmentation of the combined regions, especially for capturing tumor edge regions. Given that the regions were annotated based on morphological features rather than regional gene expressions, every region might not be separable based on their spatial gene expression. In fact, our results suggest that these tumor edge regions might share more similar expression patterns as detected by the three spatial components.

Lastly, we ran k-means clustering on each method's spatial components and compared the clusterings against the ground truth annotations for both datasets, as shown in the first columns of Figure 2 and 3. In the human brain tissue, none of the methods are able to completely delineate the L1-L5 regions; however, GraphTucker does achieve the highest ARI and correctly captures the L6 region, which only NSFH is able to capture with a similar structure, albeit not as smooth and slightly shifted. GraphTucker also achieves the highest ARI on the breast cancer dataset and has significantly smoother clusters compared to NSFH and NMF, the next two best methods. We also repeated spot clustering using sparse core tensors but observed that ARI was not improved when using less entries while still being better than all the baseline methods.

GraphTucker identifies important early developmental regions in high resolution Stereo-seq mouse embryos

To understand how GraphTucker may generalize to different datasets, we used two Stereo-seq mouse embryo datasets [17] as a case study to analyze how GraphTucker may identify known annotated regions in high-resolution data. The two embryos are from days 9.5 and 11.5 in the embryonic stage and have annotations of several important developmental regions.

We observed in both datasets that GraphTucker was able to accurately identify eleven regions in the day 9.5 embryo and ten in the day 11.5 embryo, shown in Figure 5. Several of the spatial components correlate highly to the annotated regions, for example the dermomyotome, heart, and liver in both embryos, and the neural crest and notochord in the day 9.5 embryo. Some regions, such as the brain, were captured by more than one spatial component, though we chose to present the spatial component that best captured the region as a whole. The blood vessel, connective tissue, inner ear, jaw and tooth, lung primordium, meninges, spinal cord, and surface ectoderm regions in the Day 11.5 embryo are not reported as we identified no spatial components that clearly matched to these regions.

Runtime analysis shows GraphTucker is scalable to Visium and Stereo-seq data

Empirical runtimes were measured on a server with a configuration of Intel® Xeon® E52687W v3 3.10GHz, 25M Cache, and 256GB of memory. We measured the time needed for 50 iterations on each of the datasets. We report the average time needed for one iteration, and do not observe any significant deviance at later iterations. A full table of ranks used for runtime testing are provided in Table S6. The runtime for each method across each dataset are provided in Figure S5.

Running GraphTucker on the four Visium datasets and MOSTA 9.5 dataset took between one to two hours to finish 1000 iterations using around 20GB memory. Although GraphTucker was run using 5000 maximum iterations to ensure sufficient convergence in our experiments, we observe similar results for spatial components when running with 1000 iterations that produces visually identical spatial components in significantly reduced total runtime.

GraphTucker and the MATLAB NMF implementation are both several times slower than FIST and RNMTF on both Visium and Stereo-seq data. Since runtime is significantly dominated by the n_g term, with relatively low ranks r_x, r_y, and r_g, runtime is not significantly affected by the size of the tissue n_x and n_y unless using very high resolution spatial transcriptomics data. We observe this substantial increase on

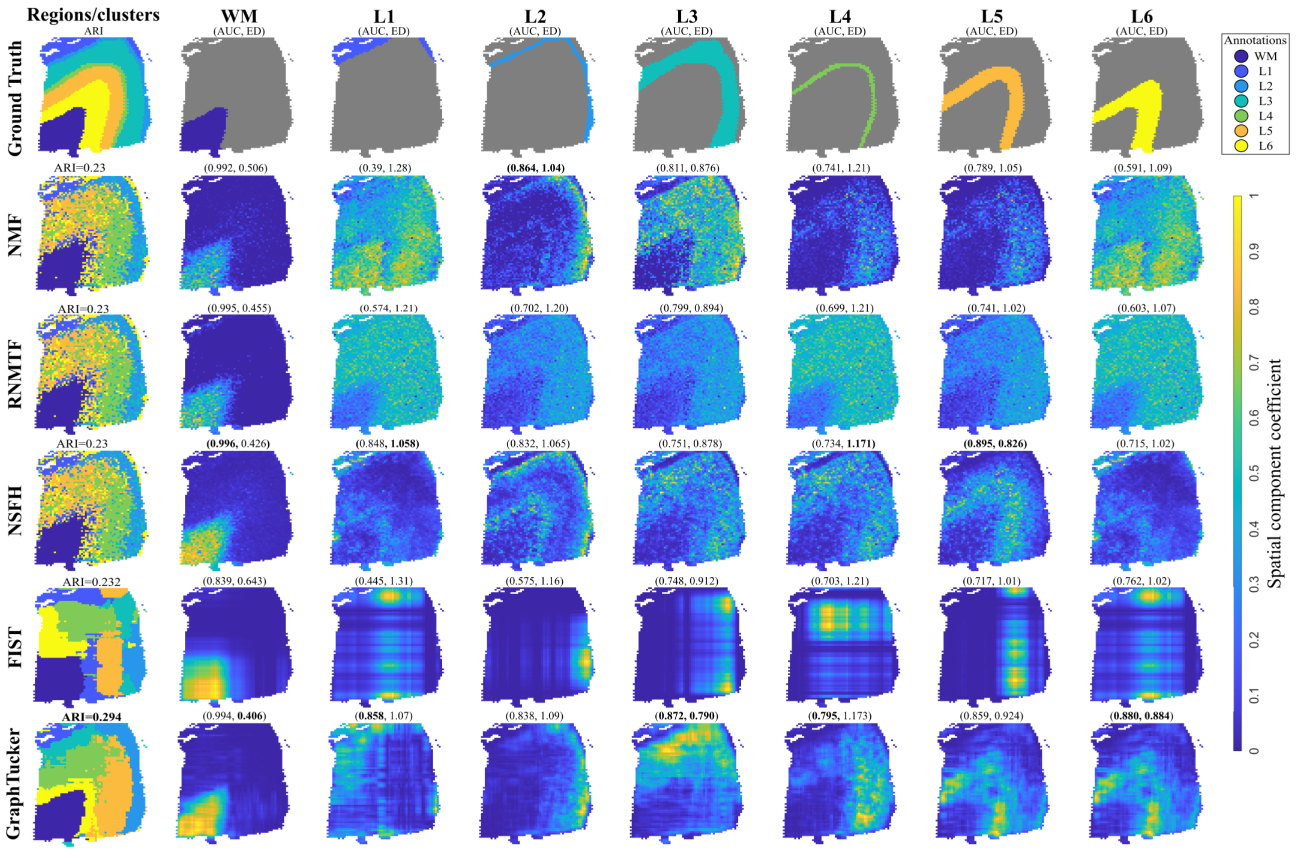

Fig. 2. Comparison of seven annotated regions from the Visium human brain dataset with matched spatial components. Area Under the receiving operator Curve (AUC) and Euclidean Distance (ED) between spatial component and region annotation is listed above each component. The clustering shown for GraphTucker used all core tensor entries, and the spatial components shown used only the top 10% of entries. Best scores are marked in bold.

Table 3. Region-spatial component matching. The best metrics are marked in bold. GraphTucker$^{n\%}$ indicates using $n\%$ sparse core tensor.

Human Brain (7 regions)			BRCA1 (20 regions)		
Methods	Avg. AUC	Avg. ED	Methods	Avg. AUC	Avg. ED
NMF rank=7	0.740	1.007	NMF rank=20	0.865	1.073
RNMTF rank=(7,50)	0.734	1.007	RNMTF rank=(20,50)	0.817	1.146
NSFH L=7	0.825	0.921	NSFH L=20	0.862	1.033
FIST rank=7, λ=1	0.684	1.038	FIST rank=50, λ=1	0.791	1.033
GraphTucker rank=(30,30,7), λ=1	0.826	0.926	GraphTucker rank=(30,30,20), λ=1	0.893	1.008
GraphTucker$^{10\%}$ rank=(30,30,7), λ=1	**0.871**	**0.906**	GraphTucker$^{5\%}$ rank=(30,30,20), λ=1	**0.903**	**0.976**

the Stereo-seq Day 11.5 mouse embryo, as running GraphTucker with rank= $(64, 64, 64)$, $\lambda = 1$ on the dataset with 1000 iterations took 6 hours using 81GB memory. This large increase also occurs in NMF as shown in Figure S5.

NSFH was not directly comparable as we observed a high variance in its runtimes and numerical stability. NSFH was tested on the BRCA1 dataset with maximum inducing points and $L = 20$, and ran five times using 2000 or 10000 genes. Run times in both scenarios were inconsistent and varied between 2 to 5 hours. In comparison, the run time for GraphTucker on BRCA1 with rank=$(50, 50, 20)$, $\lambda = 0.1$ for 1000 iterations is around 1.5 hours. Thus, the runtime of NSFH and GraphTucker is similar on the Visium data.

Discussion

In the experiments, we demonstrated GraphTucker as not only an improved imputation method, but also as a high-order factorization method for finding spatial components that delineate complex spatial regions across tissues. These components are able to capture highly irregular regional structures due to their multilinear relationships and smoothness from PPI and spatial graph regularization. A significant aspect of GraphTucker shown in the spatial component analysis is its ability to identify the most important component interactions, as we observed that selecting only a top fraction of core tensor entries can lead to improved performance for spatial domain detection. This suggests that the majority of spatial variation in gene expression can be captured with a few components, and considering too many interactions may be detrimental. The option of investigating the sparse structure in its core tensor offers flexibility in identifying the most important interactions, depending on the analysis.

In our case study on two Stereo-seq mouse embryo, we sought to understand how GraphTucker can generalize on higher resolution datasets. Despite the lack of ground truth annotation, we were still able to manually identify several

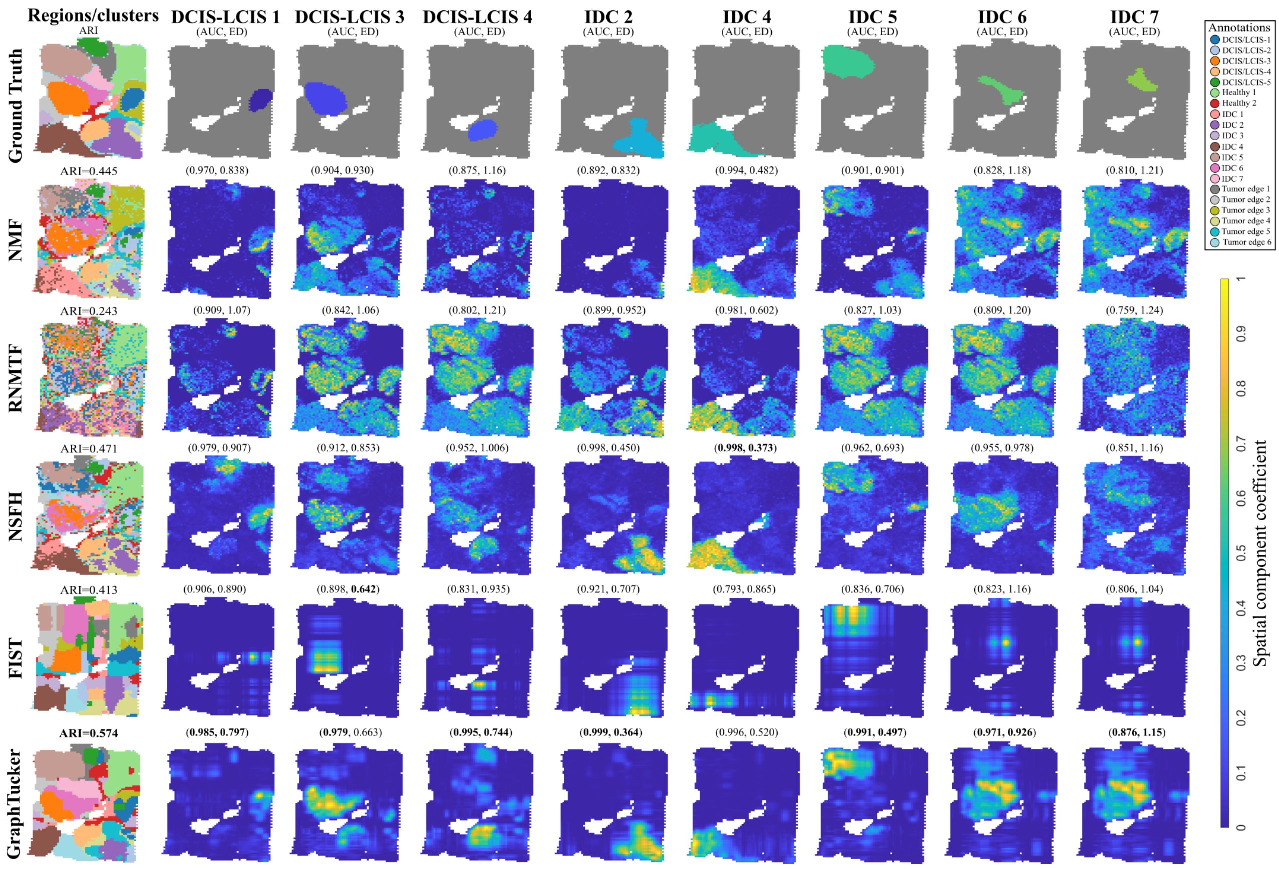

Fig. 3. Comparison of eight annotated regions from human breast cancer dataset (BRCA1) with matched spatial components. Area Under the receiving operator Curve (AUC) and Euclidean Distance (ED) between spatial component and region annotation are listed above each component. The clustering shown for GraphTucker used all core tensor entries, and the spatial components shown used only the top 5% of entries. Best scores are marked in bold.

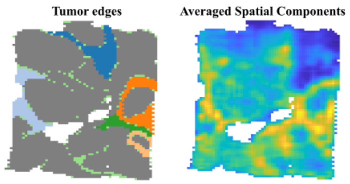

Fig. 4. Comparison of six tumor edge regions with spatial components found by GraphTucker. The combined component was averaged from three spatial components, one of which matched to four tumor edge regions.

regional matches among GraphTucker's spatial components. Several spatial components uniquely highlight certain regions, such as the heart and liver in both embryos, suggesting that the corresponding gene components are highly specific to these developmental regions.

Conclusion

GraphTucker is a novel, high-order, multilinear method for spatial transcriptomics data factorization that employs a graph-regularized Tucker decomposition model for gene expression imputation and gene and spatial component analysis. Cross-validation experiments across three Visium datasets demonstrate GraphTucker's improvement in imputation over existing methods. Visualization and quantitative analysis of GraphTucker's components on annotated human brain and human breast cancer tissues further exemplify the correctness and significance of its spatial components. Lastly, our case study on two mouse embryo datasets show potential for GraphTucker's use for generalization and downstream analysis of gene components. Further research should be conducted to analyze GraphTucker gene components as they may contain important gene modules that can reveal new cell and gene functionalities corresponding to their regional locations as indicated in the spatial components. In addition, Further application of GraphTucker on 3D datasets once they can be generated could provide even more insight into the spatial distribution of cells and their gene expressions in full 3D space.

Acknowledgments

This work is supported by fund from the National Science Foundation (NSF: #2042159).

References

1. Michaela Asp, Joseph Bergenstråhle, and Joakim Lundeberg. Spatially Resolved Transcriptomes—Next Generation Tools for Tissue Exploration. *BioEssays*, 42(10):1900221, October 2020.
2. Patrik L. Ståhl, Fredrik Salmén, and Sanja Vickovic et al. Visualization and analysis of gene expression in tissue sections by spatial transcriptomics. *Science*, 353(6294):78–82, July 2016.
3. Joseph Bergenstråhle, Ludvig Larsson, and Joakim Lundeberg. Seamless integration of image and molecular analysis for spatial transcriptomics workflows. *BMC genomics*, 21:1–7, 2020.

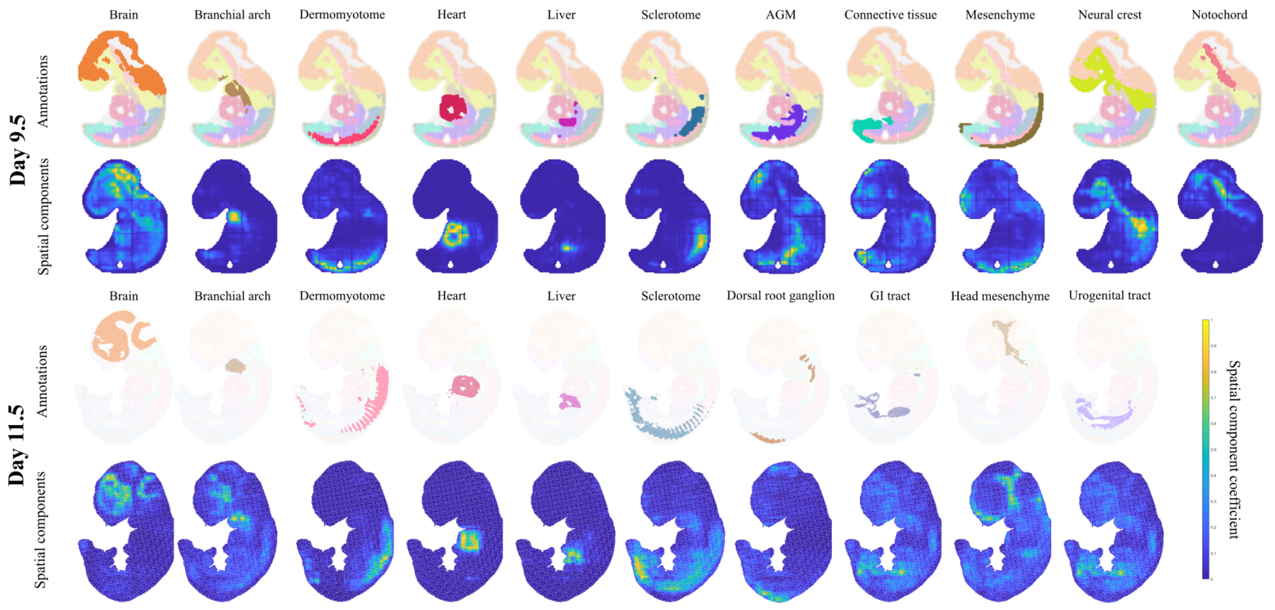

Fig. 5. Developmental regions across Days 9.5 and 11.5 mouse embryo tissue and matched spatial components from GraphTucker. Regions in the Day 11.5 embryo with no clear matching component are not shown.

4. F. William Townes and Barbara E. Engelhardt. Nonnegative spatial factorization applied to spatial genomics. *Nature Methods*, 20(2):229–238, 2024. Number: 2 Publisher: Nature Publishing Group.
5. Zhuliu Li, Tianci Song, and Jeongsik Yong et al. Imputation of spatially-resolved transcriptomes by graph-regularized tensor completion. *PLOS Computational Biology*, 17(4):e1008218, April 2021.
6. Tianci Song, Charles Broadbent, and Rui Kuang. GNTD: reconstructing spatial transcriptomes with graph-guided neural tensor decomposition informed by spatial and functional relations. *Nature Communications*, 14(1):8276, December 2023.
7. Kangning Dong and Shihua Zhang. Deciphering spatial domains from spatially resolved transcriptomics with an adaptive graph attention auto-encoder. *Nature Communications*, 13(1):1739, December 2022.
8. Tommaso Biancalani, Gabriele Scalia, and Lorenzo Buffoni et al. Deep learning and alignment of spatially resolved single-cell transcriptomes with Tangram. *Nature Methods*, 18(11):1352–1362, November 2021.
9. Bryan He, Ludvig Bergenstråhle, and Linnea Stenbeck et al. Integrating spatial gene expression and breast tumour morphology via deep learning. *Nature Biomedical Engineering*, 4(8):827–834, August 2020.
10. Yuansong Zeng, Zhuoyi Wei, and Weijiang Yu et al. Spatial transcriptomics prediction from histology jointly through Transformer and graph neural networks. *Briefings in Bioinformatics*, 23(5):bbac297, September 2022.
11. Chen Shengquan, Zhang Boheng, and Chen Xiaoyang et al. stPlus: a reference-based method for the accurate enhancement of spatial transcriptomics. *Bioinformatics*, 37(Supplement_1):i299–i307, August 2021.
12. 10x Genomics. Visium spatial transcriptomics, www.10xgenomics.com/products/spatial-gene-expression, 2019.
13. Hang Xu, Huazhu Fu, and Yahui Long et al. Unsupervised spatially embedded deep representation of spatial transcriptomics. *Genome Medicine*, 16(1):12, January 2024.
14. Brenda Pardo, Abby Spangler, and Lukas M. Weber et al. spatialLIBD: an R/Bioconductor package to visualize spatially-resolved transcriptomics data. *BMC Genomics*, 23(1):434, June 2022.
15. Kristen R. Maynard, Leonardo Collado-Torres, and Lukas M. Weber et al. Transcriptome-scale spatial gene expression in the human dorsolateral prefrontal cortex. *Nature Neuroscience*, 24(3):425–436, March 2021. Number: 3 Publisher: Nature Publishing Group.
16. Louise Huuki-Myers, Abby Spangler, and Nick Eagles et al. Integrated single cell and unsupervised spatial transcriptomic analysis defines molecular anatomy of the human dorsolateral prefrontal cortex. *bioRxiv: The Preprint Server for Biology*, page 2023.02.15.528722, February 2023.
17. Ao Chen, Sha Liao, and Mengnan Cheng et al. Spatiotemporal transcriptomic atlas of mouse organogenesis using DNA nanoball-patterned arrays. *Cell*, 185(10):1777–1792.e21, May 2022.
18. Chris Stark, Bobby-Joe Breitkreutz, and Teresa Reguly et al. BioGRID: a general repository for interaction datasets. *Nucleic Acids Research*, 34(Database issue):D535–D539, January 2006.
19. Michael W Berry, Murray Browne, and Amy N Langville et al. Algorithms and applications for approximate nonnegative matrix factorization. *Computational statistics & data analysis*, 52(1):155–173, 2007.
20. TaeHyun Hwang, Gowtham Atluri, and MaoQiang Xie et al. Co-clustering phenome–genome for phenotype classification and disease gene discovery. *Nucleic Acids Research*, 40(19):e146–e146, 06 2012.
