# Supplementary material for: Deciphering high-order structures in spatial transcriptomes with graph-guided Tucker decomposition
Source: Bioinformatics. 2024 Jun 28;40(Suppl 1):i529–38. doi: 10.1093/bioinformatics/btae245 (PMC11256919; doi:10.1093/bioinformatics/btae245)
Supplement: btae245_Supplementary_Data [file btae245_supplementary_data.zip › btae245_Supplementary_Data/Kuang.108.sup.1.pdf]

S1 Preliminaries

Table 1: **Notations.**

Operand	Operator
**Scalar:** x	**Hadamard product:** $\odot$
**Vector:** $\boldsymbol{x}$	**Kronecker sum:** $\oplus$
**Matrix:** $\mathbf{X}$	**Kronecker product:** $\otimes$
**Tensor :** $\boldsymbol{\mathsf{X}}$	**Vector outer product:** $\circ$
**Vectorization of a tensor:** $\text{vec}(\boldsymbol{\mathsf{X}})$	n**-mode product of a tensor:** $\times_n$
**Mode-**n **matricization of a tensor:** $\mathbf{X}_{(n)}$	**vec-transpose of a matrix:** $\mathbf{X}^{(p)}$

S1.1 Tensor Completion

In situ capturing (ISC) methods measure gene expression at different locations using capturing probes arranged in a spatial array over cell populations or tissues, and spatial gene expressions can be naturally represented by a tensor, with dimensions corresponding to genes and different spatial axes. Consequently, the primary challenge of reconstructing incomplete spatial gene expression can be easily formulated as a tensor completion problem, which aims to impute missing or unobserved entries of a partially observed tensor by learning its representation with decomposition. In this section, we will first introduce the basic concepts of tensors and mathematically define the tensor completion problem. And lastly, we will briefly review the state-of-the-art tensor decomposition models for tensor completion.

S1.1.1 Tensor Basics

Tensor. A tensor is generally defined as a multidimensional array, with its dimensionality described as order and each specific dimension termed as a mode. An M-order tensor is also known as an M-way or mode-M tensor, often denoted as $\boldsymbol{\mathsf{T}} \in \mathbb{R}^{n_1 \times n_2 \times \ldots \times n_M}$, where n_m represents the number of entries in the respective m-th mode. For example, the spatial gene expressions is a typical 3-way tensor $\boldsymbol{\mathsf{T}} \in \mathbb{R}_+^{n_g \times n_x \times n_y}$, where the entry $\boldsymbol{\mathsf{T}}_{i_g i_x i_y}$ at the index (i_g, i_x, i_y) indicates the value for i_g-th gene expressed at the location (i_x, i_y).

Rank-one Tensor. An M-way tensor is called a rank-one tensor if it can be strictly decomposed into the outer product of M vectors.

$$\boldsymbol{\mathsf{T}} = \mathbf{a}_1 \circ \mathbf{a}_2 \circ \ldots \circ \mathbf{a}_M, \boldsymbol{\mathsf{T}}_{i_1 i_2 \ldots i_M} = [\mathbf{a}_1]_{i_1} [\mathbf{a}_2]_{i_2} \ldots [\mathbf{a}_M]_{i_M} \tag{1}$$

where M column vectors $\mathbf{a}_1 \in \mathbb{R}^{n_1}, \mathbf{a}_2 \in \mathbb{R}^{n_2}, \ldots, \mathbf{a}_N \in \mathbb{R}^{n_M}$, and $\circ$ denotes the vector outer product.

Tensor Rank. The definition of tensor rank varies depending on the learning tasks. There are two primary definitions of tensor rank. In the first definition,

the rank of a tensor $\boldsymbol{\mathcal{T}}$ denotes the minimum number of rank-one tensors required to generate $\boldsymbol{\mathcal{T}}$ by summation, which can be expressed as:

$$\operatorname{rank}(\boldsymbol{\mathcal{T}}) = \min\{R \in Z_+ : \exists\{\mathbf{a}_m^r\}, s.t. \boldsymbol{\mathcal{T}} = \sum_{r=1}^{R} \mathbf{a}_1^r \circ \mathbf{a}_2^r \circ \ldots \circ \mathbf{a}_M^r\} \tag{2}$$

Rather than summarizing the tensor rank into one scalar, the second definition simply describes the rank of a tensor $\boldsymbol{\mathcal{T}}$ as a set of ranks of the tensor matricizations along different modes, which can be represented as follows:

$$\operatorname{rank}(\boldsymbol{\mathcal{T}}) = \{\operatorname{rank}(\boldsymbol{\mathcal{T}}_{(1)}), \operatorname{rank}(\boldsymbol{\mathcal{T}}_{(2)}), \ldots, \operatorname{rank}(\boldsymbol{\mathcal{T}}_{(M)})\} \tag{3}$$

Here, $\boldsymbol{\mathcal{T}}_{(m)}$ denotes the tensor matricization, which unfolds a tensor $\boldsymbol{\mathcal{T}} \in \mathbb{R}^{n_1 \times n_2 \times \ldots \times n_M}$ along its m-th mode to a matrix $\boldsymbol{\mathcal{T}}_{(m)} \in \mathbb{R}^{n_m \times \prod_{k \neq m} n_k}, \forall m \in \{1, 2, \ldots, M\}$, $\operatorname{rank}(\boldsymbol{\mathcal{T}}_{(m)})$ indicates the column rank of the matrix $\boldsymbol{\mathcal{T}}_{(m)}$.

S1.1.2 Tensor Completion Definition

Tensor completion involves inferring the missing entries based on a partially observed tensor. To avoid being an underdetermined and intractable problem, a common approach is to assume the low-rank property in the tensor, which helps constrain the degrees of freedom of the missing entries. Based on the definitions above, given a tensor $\boldsymbol{\mathcal{T}}$ with missing entries, the tensor completion can be mathematically formulated as follows:

$$\begin{aligned} &\min_{\hat{\boldsymbol{\mathcal{T}}}} \operatorname{rank}(\hat{\boldsymbol{\mathcal{T}}}) \\ &s.t. \quad \|\boldsymbol{\mathcal{M}} \odot (\boldsymbol{\mathcal{T}} - \hat{\boldsymbol{\mathcal{T}}})\|_F^2 = 0 \end{aligned} \tag{4}$$

Here, $\odot$ denotes the Hadamard product, which is the element-wise product of two tensors with the same size. $\hat{\boldsymbol{\mathcal{T}}}$ represents the imputed tensor, $\boldsymbol{\mathcal{M}} \in \{0, 1\}^{n_1 \times n_2 \times \ldots \times n_M}$ represents the mask tensor indicating observed entries in the $\boldsymbol{\mathcal{T}}$, and $\boldsymbol{\mathcal{M}}_{i_1 i_2 \ldots i_M}$ is set to be 1 if the entry at the index $(i_1, i_2, \ldots, i_M)$ is observed in $\boldsymbol{\mathcal{T}}$, and 0 otherwise.

While it seems intuitive to define the task by seeking a tensor $\hat{\boldsymbol{\mathcal{T}}}$ with the minimum rank and subjecting to the equality constraints based on observations, solving this optimization problem has been proven to be NP-hard. To reduce the complexity of the problem, a feasible alternative is to fix the tensor rank and substitute the objective function with approximating the imputed tensor in Eq. 4, which is the same optimization problem in tensor decomposition, the tensor completion can be rewritten as follows:

$$\begin{aligned} &\min_{\hat{\boldsymbol{\mathcal{T}}}} \|\boldsymbol{\mathcal{M}} \odot (\boldsymbol{\mathcal{T}} - \hat{\boldsymbol{\mathcal{T}}})\|_F^2 \\ &s.t. \quad \operatorname{rank}(\hat{\boldsymbol{\mathcal{T}}}) = R \end{aligned} \tag{5}$$

S1.1.3 Tensor Decomposition Models

Given the computational expense and impracticality of analyzing or storing dense tensors, particularly in high-order or large-scale scenarios, the primary goal of tensor decomposition is to learn a compressed representation. Tensor decomposition can serve as an intermediate step to solve the tensor completion problem by leveraging its inherent low-rank property in the compressed representation. In this section, we will briefly review two classical tensor decomposition models that have been widely adopted for tensor completion.

CANDECOMP/PARAFAC (CP) Decomposition. The key idea of the CP decomposition is to express a tensor as the sum of a finite number of rank-one tensors. Given an M-way tensor $\boldsymbol{\mathcal{T}}$, the CP decomposition can be formalized as follows:

$$\begin{aligned} &\min_{\hat{\boldsymbol{\mathcal{T}}}} \|\boldsymbol{\mathcal{M}} \odot (\boldsymbol{\mathcal{T}} - \hat{\boldsymbol{\mathcal{T}}})\|_F^2 \\ &s.t. \quad \hat{\boldsymbol{\mathcal{T}}} = [\![\mathbf{A}_1, \mathbf{A}_2, \ldots, \mathbf{A}_M]\!] \end{aligned} \tag{6}$$

Here, $[\![\cdot]\!]$ denotes the Kruskal operator, where $[\![\mathbf{A}_1, \mathbf{A}_2, \ldots, \mathbf{A}_M]\!] = \sum_{r=1}^{R} \mathbf{a}_1^r \circ \mathbf{a}_2^r \circ \ldots \circ \mathbf{a}_M^r$, $\mathbf{A_m} \in \mathbb{R}^{n_m \times R}$ represents the factor matrix along mode-m, and $\mathbf{a}_m^r$ is the r-th column of $\mathbf{A_m}$.

Tucker Decomposition. The Tucker decomposition is often considered as a form of higher-order principal component analysis. The key idea is to decompose a tensor into a core tensor transformed by matrices along different modes. Given an M-way tensor $\boldsymbol{\mathcal{T}}$, the Tucker decomposition can be defined as follows:

$$\begin{aligned} &\min_{\hat{\boldsymbol{\mathcal{T}}}} \|\boldsymbol{\mathcal{M}} \odot (\boldsymbol{\mathcal{T}} - \hat{\boldsymbol{\mathcal{T}}})\|_F^2 \\ &s.t. \quad \hat{\boldsymbol{\mathcal{T}}} = [\![\boldsymbol{\mathcal{G}}; \mathbf{A}_1, \mathbf{A}_2, \ldots, \mathbf{A}_M]\!] \end{aligned} \tag{7}$$

Here, $\boldsymbol{\mathcal{G}} \in \mathbb{R}^{R_1 \times R_2 \times \ldots \times R_M}$ denotes the core tensor, and $\mathbf{A_m} \in \mathbb{R}^{n_m \times R_m}$ represents the factor matrix along mode-m.

Tucker decomposition offers greater flexibility than CP decomposition under the same rank assumption. This is because Tucker decomposition incorporates every possible interaction between the factor matrices across different modes by introducing the core tensor, allowing it to capture more complex interactions that may not be strictly multilinear. However, Tucker decomposition is also more prone to overfitting, especially when the tensor is sparse, due to the potentially overly complex model compared to CP decomposition in the same rank setting.

S1.2 Regularization with Product Graph

Spatial relations among capturing probes in ISC methods are crucial prior knowledge to sketch the transcriptional landscape within cell populations or tissues, as capturing probes in the vicinity tend to exhibit similar gene expressions. Similarly, functional relations among genes in protein-protein interactions (PPIs) are

vital prior knowledge to abstract the biological activities within cell populations or tissues, as genes involved in the same interactions are more likely to show coherent expression patterns. Both these spatial and functional relations can be easily encoded in graphs, where capturing probes or genes denote nodes while edges connecting nodes represent their corresponding relations. To integrate the prior knowledge in spatial and gene relations, we introduce product graph regularization to incorporate their graphs to guide the learning tasks. In this section, we will review a few varieties of product graphs, and then formally define the product graph regularization employed in GraphTucker.

S1.2.1 Product Graph

Given M undirected graphs $\{G_m = (V_m, E_m) : m = 1, 2, \ldots, M\}$, where V_m and E_m denotes the set of nodes and edges in the graph G_m, and $|V_m| = n_m$ indicates the number of nodes in the G_m. Let $\mathbf{W}_m$ be the adjacency matrix of G_m, where $[\mathbf{W}_m]_{ij} = 1$ if there is an edge between i- and j-th nodes in G_m and 0 otherwise, and $\mathbf{D}_m = \mathrm{diag}\,(d_1, \ldots, d_{n_m}) \in \mathbb{Z}^{n_m \times n_m}$ be the degree matrix of G_m with $d_{ij} = \sum_j [\mathbf{W}_m]_{ij}$, where . Then $\mathbf{L}_m = \mathbf{D}_m - \mathbf{W}_m \in \mathbf{R}^{n_m \times n_m}$ represents the graph Laplacian for G_m.

The product on a set of undirected graphs $\{G_m, m = 1, 2, \ldots, M\}$ is an operation that combines them into a new undirected graph, known as product graph, $G_p = (V_p, E_p)$ with the number of nodes $|V_p| = \prod_{m=1}^{M} |V_m|$. For any pair of nodes $(a_1, a_2, \ldots, a_M)$ and $(b_1, b_2, \ldots, b_M)$ in the product graph G_p, the corresponding edge is determined by the adjacency or equality of a_m and b_m in the graph G_m. There are three main types of product graphs distinguished by how edges are determined:

Cartesian Product Graph. A pair of nodes $(a_1, a_2, \ldots, a_M)$ and $(b_1, b_2, \ldots, b_M)$ in the Cartesian product graph G_c is adjacent if and only if any pair of nodes a_m and b_m is adjacent in G_m while the remaining pairs of nodes a_k and b_k are identical in the corresponding graph $G_k, \forall k \neq m$. The adjacency matrix and Laplacian matrix of G_c can be calculated by $\mathbf{W}_c = \oplus_{m=1}^{M} \mathbf{W}_m$ and $\mathbf{L}_c = \oplus_{m=1}^{M} \mathbf{L}_m$ respectively, where $\oplus$ denotes the Kronecker sum.

Tensor Product Graph. A pair of nodes $(a_1, a_2, \ldots, a_M)$ and $(b_1, b_2, \ldots, b_M)$ in the tensor product graph G_t is adjacent if and only if every pair of nodes a_m and b_m is adjacent in the corresponding $G_m, \forall k \neq m$. The adjacency matrix and Laplacian matrix of G_{tp} can be calculated by $\mathbf{W}_p = \otimes_{m=1}^{M} \mathbf{W}_m$ and $\mathbf{L}_t = \otimes_{m=1}^{M} \mathbf{D}_m - \otimes_{m=1}^{M} \mathbf{W}_m$ respectively, where $\otimes$ denotes the Kronecker product.

Strong Product Graph. A pair of nodes $(a_1, a_2, \ldots, a_M)$ and $(b_1, b_2, \ldots, b_M)$ in the strong product graph G_s is adjacent if and only if is adjacent either in G_c or G_t. The adjacency matrix and Laplacian matrix of G_s can be calculated by $\mathbf{W}_s = \mathbf{W}_c + \mathbf{W}_t$ and $\mathbf{L}_s = \mathbf{L}_c + \mathbf{L}_t$ respectively.

According to the above definitions, tensor and strong product graphs apparently have more edges compared with the Cartesian product graph, which implies that tensor and strong product graphs consider more combinations among nodes as potential interactions in G_p; however, they might also impose strong assumptions regarding similarities among nodes and introduce additional noises to the learning problem that leveraging the prior knowledge in the G_p.

S1.2.2 Product Graph Laplacian Regularization

Let G_p be one of the product graphs defined above, combining the set of graphs $\{G_m : m = 1, 2, \ldots, M\}$, where edges indicate similarities among nodes. $\mathbf{W}_p$ and $\mathbf{L}_p$ represent the adjacency matrix and Laplacian matrix of G_p respectively. Suppose every node in G_p is associated with a value, where some nodes are assigned zeros to indicate unknown values. These values can be concatenated into a vector $\mathbf{x} \in \mathbb{R}^{|V_p|}$. The general learning problem is to produce a new vector $\hat{\mathbf{x}} \in \mathbb{R}^{|V_p|}$ with unknown values estimated by utilizing the prior knowledge encoded in the Laplacian regularization $\mathbf{L}_p$. The idea of Laplacian regularization is to smooth $\hat{\mathbf{x}}$ over the manifolds of the graph G_p, ensuring that adjacent nodes share similar values. For instance, given spatial gene expression, Laplacian regularization of the product graph of protein-protein interaction (PPI) and spatial graphs encourages expression values to be similar if the genes are functionally related or capturing probes are spatially adjacent. The Laplacian regularization of the product graph can be mathematically defined in the quadratic form: $\hat{\mathbf{x}}^T \mathbf{L}_p \hat{\mathbf{x}} = \sum_{ij} [\mathbf{W}_p]_{ij} (\hat{\mathbf{x}}_i - \hat{\mathbf{x}}_j)$. The general learning problem with product graph Laplacian regularization can be formulated as follows:

$$\min_{\hat{\mathbf{x}}} \| m \odot (\mathbf{x} - \hat{\mathbf{x}}) \|_2^2 + \lambda \hat{\mathbf{x}}^T \mathbf{L}_p \hat{\mathbf{x}} \tag{8}$$

where $m \in \{0, 1\}^{|V_p|}$ indicates nodes in G_p with known values, and $\lambda > 0$ represents an appropriately chosen regularization parameter, which indicates the strength of prior knowledge in the product graph introduced in the learning.

It is straightforward to incorporate product graph Laplacian regularization in the tensor completion we defined in the section S1.1, and the learning problem was reformulated in the work [**?**] as follows:

$$\min_{\hat{\boldsymbol{T}}} \| \boldsymbol{M} \odot (\boldsymbol{T} - \hat{\boldsymbol{T}}) \|_F^2 + \lambda \mathrm{vec}(\hat{\boldsymbol{T}})^T \mathbf{L}_p \mathrm{vec}(\hat{\boldsymbol{T}}) \tag{9}$$

where $\mathrm{vec}(\cdot)$ denotes the function reshaping the tensor into a vector matching the order of nodes in G_p.

S1.3 Important definitions

S1.3.1 Vec-transpose

The vec-transpose (p) is a matrix operator the generalizes the transpose, and is necessary for formulating derivatives of functions that use the Kronecker product.

Given a matrix $\mathbf{X} \in \mathbb{R}^{(r \times c)}$, p is an integer that evenly divides the number of rows r, and its usage can be best understood by observing the following properties:

$$\begin{aligned}\mathbf{X}^{(1)} &= \mathbf{X}^T \\ (\mathbf{X}^{(p)})^{(p)} &= \mathbf{X} \\ \mathbf{X}^{(r)} &= \mathbf{vec}(\mathbf{X}) \\ \mathbf{vec}(\mathbf{X})^{(p)} &= \mathrm{reshape}(\mathbf{vec}(\mathbf{X}), rp, c/p)\end{aligned} \tag{10}$$

Where `reshape()` is equivalent to the MATLAB function of the same name.

S1.3.2 Relationship between the Kronecker and n-mode products

A mode-n unfolding of a tensor $\boldsymbol{\mathcal{X}}$ constructed using the Kronecker product in the following form can be calculated as a tensor using n-mode product as follows:

$$\begin{aligned}&\mathbf{X}_{(n)} = \mathbf{A}^{(n)}\mathbf{G}_{(n)}(\mathbf{A}^{(N)} \otimes \cdots \otimes \mathbf{A}^{(n+1)} \otimes \mathbf{A}^{(n-1)} \otimes \cdots \otimes \mathbf{A}^{(1)})^T \\ &\qquad\qquad\qquad\qquad \Updownarrow \\ &\boldsymbol{\mathcal{X}} = \boldsymbol{\mathcal{G}} \times_1 \mathbf{A}^{(1)} \times_2 \mathbf{A}^{(2)} \cdots \times_N \mathbf{A}^{(N)}\end{aligned} \tag{11}$$

This property is extremely useful for computing a series of Kronecker products, as using the n-mode product in this manner requires less overall operations.

S2 Graph regularized nonnegative Tucker decomposition optimization by multiplicative updating

A local optimal solution is computed by GraphTucker using a multiplicative update (MU) approach performing multiplicative updates on the factor matrices and core tensor. Here, we show the update rules with respect to $\mathbf{A}_g$ without loss of generality for the update rules of $\mathbf{A}_x$ and $\mathbf{A}_y$.

First, the objective function can be rewritten as follows:

$$\begin{aligned}\mathcal{F} &= \mathcal{F}_1 + \lambda\mathcal{F}_2 \\ \mathcal{F}_1 &= \frac{1}{2}||\boldsymbol{\mathcal{M}} \odot (\boldsymbol{\mathcal{T}} - [\![\boldsymbol{\mathcal{G}}; \mathbf{A}_y, \mathbf{A}_x, \mathbf{A}_g]\!])||_F^2 \\ \mathcal{F}_2 &= \frac{\lambda}{2}\mathrm{vec}([\![\boldsymbol{\mathcal{G}}; \mathbf{A}_y, \mathbf{A}_x, \mathbf{A}_g]\!])^T \mathbf{L}_c \mathrm{vec}([\![\boldsymbol{\mathcal{G}}; \mathbf{A}_y, \mathbf{A}_x, \mathbf{A}_g]\!])\end{aligned} \tag{12}$$

The partial derivative of $\mathcal{F}_1$ with respect to $\mathbf{A}_g$ can be calculated as:

$$\frac{\partial \mathcal{F}_1}{\partial \mathbf{A}_g} = (\mathbf{M}_{(g)} \odot \hat{\mathbf{T}}_{(g)} - \mathbf{M}_{(g)} \odot \mathbf{T}_{(g)})(\mathbf{G}_{(g)}(\mathbf{A}_x \otimes \mathbf{A}_y)^T)^T \tag{13}$$

where $\cdot_{(g)}$ is the g-mode unfolding of a tensor.

For the second term of the objective function, the partial derivative of $\mathcal{F}_2$ with respect to $\mathbf{A}_g$ can be calculated as:

$$\begin{aligned}\frac{\partial \mathcal{F}_2}{\partial \mathbf{A}_g} &= \mathbf{A}_g(\mathbf{vec}(\mathbf{G}_{(y)})^{(p)})^T(\mathbf{A}_x^T\mathbf{A}_x \otimes \mathbf{A}_y^T\mathbf{L}_y\mathbf{A}_y)\mathbf{vec}(\mathbf{G}_{(y)})^{(p)} \\ &+ \mathbf{A}_g(\mathbf{vec}(\mathbf{G}_{(y)})^{(p)})^T(\mathbf{A}_x^T\mathbf{L}_x\mathbf{A}_x \otimes \mathbf{A}_y^T\mathbf{A}_y)\mathbf{vec}(\mathbf{G}_{(y)})^{(p)} \\ &+ \mathbf{L}_g\mathbf{A}_g(\mathbf{vec}(\mathbf{G}_{(y)})^{(p)})^T(\mathbf{A}_x^T\mathbf{A}_x \otimes \mathbf{A}_y^T\mathbf{A}_y)\mathbf{vec}(\mathbf{G}_{(y)})^{(p)}.\end{aligned} \tag{14}$$

In order to update the entries of $\mathbf{A}_g$ using a multiplicative update, we must separate the positive and negative components from each partial derivative $\frac{\partial \mathcal{F}_1}{\partial \mathbf{A}_g}$ $\frac{\partial \mathcal{F}_2}{\partial \mathbf{A}_g}$ as follows:

$$\begin{aligned}\mathcal{F} &= \mathcal{F}_1 + \lambda\mathcal{F}_2 \\ \frac{\partial \mathcal{F}}{\partial \mathbf{A}_g} &= \frac{\partial \mathcal{F}_1}{\partial \mathbf{A}_g} + \lambda\frac{\partial \mathcal{F}_2}{\partial \mathbf{A}_g} \\ &= \left[\frac{\partial \mathcal{F}_1}{\partial \mathbf{A}_g}\right]^+ - \left[\frac{\partial \mathcal{F}_1}{\partial \mathbf{A}_g}\right]^- + \lambda\left(\left[\frac{\partial \mathcal{F}_2}{\partial \mathbf{A}_g}\right]^+ - \left[\frac{\partial \mathcal{F}_2}{\partial \mathbf{A}_g}\right]^-\right)\end{aligned} \tag{15}$$

where $\left[\frac{\partial \mathcal{F}_1}{\partial \mathbf{A}_g}\right]^+$ and $\left[\frac{\partial \mathcal{F}_1}{\partial \mathbf{A}_g}\right]^-$ are the positive and negative components of $\frac{\partial \mathcal{F}_1}{\partial \mathbf{A}_g}$ and are equal to:

$$\begin{aligned}\left[\frac{\partial \mathcal{F}_1}{\partial \mathbf{A}_g}\right]^+ &= (\mathbf{M}_{(g)} \odot \hat{\mathbf{T}}_{(g)}(\mathbf{G}_{(g)}(\mathbf{A}_x \otimes \mathbf{A}_y)^T)^T \\ \left[\frac{\partial \mathcal{F}_1}{\partial \mathbf{A}_g}\right]^- &= (\mathbf{M}_{(g)} \odot \mathbf{T}_{(g)})(\mathbf{G}_{(g)}(\mathbf{A}_x \otimes \mathbf{A}_y)^T)^T\end{aligned} \tag{16}$$

The positive and negative components $\left[\frac{\partial \mathcal{F}_2}{\partial \mathbf{A}_g}\right]^+$ and $\left[\frac{\partial \mathcal{F}_2}{\partial \mathbf{A}_g}\right]^-$ for the second term can be obtained using the fact that $\mathbf{L}_g = \mathbf{D}_g - \mathbf{W}_g$:

$$\begin{aligned}\left[\frac{\partial \mathcal{F}_2}{\partial \mathbf{A}_g}\right]^+ &= \mathbf{A}_g(\mathbf{vec}(\mathbf{G}_{(y)})^{(p)})^T(\mathbf{A}_x^T\mathbf{A}_x \otimes \mathbf{A}_y^T\mathbf{D}_y\mathbf{A}_y)\mathbf{vec}(\mathbf{G}_{(y)})^{(p)} \\ &+ \mathbf{A}_g(\mathbf{vec}(\mathbf{G}_{(y)})^{(p)})^T(\mathbf{A}_x^T\mathbf{D}_x\mathbf{A}_x \otimes \mathbf{A}_y^T\mathbf{A}_y)\mathbf{vec}(\mathbf{G}_{(y)})^{(p)} \\ &+ \mathbf{D}_g\mathbf{A}_g(\mathbf{vec}(\mathbf{G}_{(y)})^{(p)})^T(\mathbf{A}_x^T\mathbf{A}_x \otimes \mathbf{A}_y^T\mathbf{A}_y)\mathbf{vec}(\mathbf{G}_{(y)})^{(p)}\end{aligned} \tag{17}$$

$$\begin{aligned}\left[\frac{\partial \mathcal{F}_2}{\partial \mathbf{A}_g}\right]^- &= \mathbf{A}_g(\mathbf{vec}(\mathbf{G}_{(y)})^{(p)})^T(\mathbf{A}_x^T\mathbf{A}_x \otimes \mathbf{A}_y^T\mathbf{W}_y\mathbf{A}_y)\mathbf{vec}(\mathbf{G}_{(y)})^{(p)} \\ &+ \mathbf{A}_g(\mathbf{vec}(\mathbf{G}_{(y)})^{(p)})^T(\mathbf{A}_x^T\mathbf{W}_x\mathbf{A}_x \otimes \mathbf{A}_y^T\mathbf{A}_y)\mathbf{vec}(\mathbf{G}_{(y)})^{(p)} \\ &+ \mathbf{W}_g\mathbf{A}_g(\mathbf{vec}(\mathbf{G}_{(y)})^{(p)})^T(\mathbf{A}_x^T\mathbf{A}_x \otimes \mathbf{A}_y^T\mathbf{A}_y)\mathbf{vec}(\mathbf{G}_{(y)})^{(p)}\end{aligned} \tag{18}$$

Using these components, we create the following multiplicative update rule for $\mathbf{A}_g$:

$$\left[\mathbf{A}_g\right]_{i,j} \longleftarrow \left[\mathbf{A}_g\right]_{i,j} \left(\frac{\left[\frac{\partial \mathcal{F}_1}{\partial \mathbf{A}_g}\right]^{-}_{i,j} + \lambda \left[\frac{\partial \mathcal{F}_2}{\partial \mathbf{A}_g}\right]^{-}_{i,j}}{\left[\frac{\partial \mathcal{F}_1}{\partial \mathbf{A}_g}\right]^{+}_{i,j} + \lambda \left[\frac{\partial \mathcal{F}_2}{\partial \mathbf{A}_g}\right]^{+}_{i,j}} \right) \tag{19}$$

Where i, j indicate the $(i, j)-$th entry in the factor matrix. The update rules for $\mathbf{A}_x$ and $\mathbf{A}_y$ can be derived similarly without loss of generality, giving the following update rules for each:

$$\left[\mathbf{A}_x\right]_{i,j} \longleftarrow \left[\mathbf{A}_x\right]_{i,j} \left(\frac{\left[\frac{\partial \mathcal{F}_1}{\partial \mathbf{A}_x}\right]^{-}_{i,j} + \lambda \left[\frac{\partial \mathcal{F}_2}{\partial \mathbf{A}_x}\right]^{-}_{i,j}}{\left[\frac{\partial \mathcal{F}_1}{\partial \mathbf{A}_x}\right]^{+}_{i,j} + \lambda \left[\frac{\partial \mathcal{F}_2}{\partial \mathbf{A}_x}\right]^{+}_{i,j}} \right) \tag{20}$$

$$\left[\mathbf{A}_y\right]_{i,j} \longleftarrow \left[\mathbf{A}_y\right]_{i,j} \left(\frac{\left[\frac{\partial \mathcal{F}_1}{\partial \mathbf{A}_y}\right]^{-}_{i,j} + \lambda \left[\frac{\partial \mathcal{F}_2}{\partial \mathbf{A}_y}\right]^{-}_{i,j}}{\left[\frac{\partial \mathcal{F}_1}{\partial \mathbf{A}_y}\right]^{+}_{i,j} + \lambda \left[\frac{\partial \mathcal{F}_2}{\partial \mathbf{A}_y}\right]^{+}_{i,j}} \right) \tag{21}$$

Lastly, we update the core tensor $\boldsymbol{\mathcal{G}}$ in a similar way, by computing the partial derivatives of each term and using the positive and negative components. Here, we compute the partial derivatives of with respect to $\mathbf{vec}(\mathbf{G}_{(g)})$ as it gives a more simplified derivation and calculation.

The partial derivative of $\mathcal{F}_1$ and $\mathcal{F}_2$ with respect to $\mathbf{vec}(\mathbf{G}_{(g)})$ can be calculated as:

$$\frac{\partial \mathcal{F}_1}{\partial \mathbf{vec}(\mathbf{G}_{(g)})} = (\mathbf{A}_x \otimes \mathbf{A}_y \otimes \mathbf{A}_g)^T \Big(\mathbf{vec}(\mathbf{M}_{(g)} \odot \hat{\mathbf{T}}_{(g)}) - \mathbf{vec}(\mathbf{M}_{(g)} \odot \mathbf{T}_{(g)}) \Big) \tag{22}$$

$$\begin{aligned} \frac{\partial \mathcal{F}_2}{\partial \mathbf{vec}(\mathbf{G}_{(g)})} &= (\mathbf{A}_x^T \mathbf{A}_x \otimes \mathbf{A}_y^T \mathbf{A}_y \otimes \mathbf{A}_g^T \mathbf{L}_g \mathbf{A}_g) \mathbf{vec}(\mathbf{G}_{(g)}) \\ &+ (\mathbf{A}_x^T \mathbf{A}_x \otimes \mathbf{A}_y^T \mathbf{L}_y \mathbf{A}_y \otimes \mathbf{A}_g^T \mathbf{A}_g) \mathbf{vec}(\mathbf{G}_{(g)}) \\ &+ (\mathbf{A}_x^T \mathbf{L}_x \mathbf{A}_x \otimes \mathbf{A}_y^T \mathbf{A}_y \otimes \mathbf{A}_g^T \mathbf{A}_g) \mathbf{vec}(\mathbf{G}_{(g)}) \end{aligned} \tag{23}$$

These can then be split into their positive and negative components as follows:

$$\left[\frac{\partial \mathcal{F}_1}{\partial \mathbf{vec}(\mathbf{G}_{(g)})}\right]^{+} = (\mathbf{A}_x \otimes \mathbf{A}_y \otimes \mathbf{A}_g)^T \mathbf{vec}(\mathbf{M}_{(g)} \odot \hat{\mathbf{T}}_{(g)}) \tag{24}$$

$$\left[\frac{\partial \mathcal{F}_1}{\partial \mathbf{vec}(\mathbf{G}_{(g)})}\right]^{-} = (\mathbf{A}_x \otimes \mathbf{A}_y \otimes \mathbf{A}_g)^T \mathbf{vec}(\mathbf{M}_{(g)} \odot \mathbf{T}_{(g)}) \tag{25}$$

$$
\begin{aligned}
\left[\frac{\partial \mathcal{F}_2}{\partial \mathbf{vec}(\mathbf{G}_{(g)})}\right]^{+} &= (\mathbf{A}_x^T\mathbf{A}_x \otimes \mathbf{A}_y^T\mathbf{A}_y \otimes \mathbf{A}_g^T\mathbf{D}_g\mathbf{A}_g)\mathbf{vec}(\mathbf{G}_{(g)}) \\
&+ (\mathbf{A}_x^T\mathbf{A}_x \otimes \mathbf{A}_y^T\mathbf{D}_y\mathbf{A}_y \otimes \mathbf{A}_g^T\mathbf{A}_g)\mathbf{vec}(\mathbf{G}_{(g)}) \\
&+ (\mathbf{A}_x^T\mathbf{D}_x\mathbf{A}_x \otimes \mathbf{A}_y^T\mathbf{A}_y \otimes \mathbf{A}_g^T\mathbf{A}_g)\mathbf{vec}(\mathbf{G}_{(g)})
\end{aligned} \tag{26}
$$

$$
\begin{aligned}
\left[\frac{\partial \mathcal{F}_2}{\partial \mathbf{vec}(\mathbf{G}_{(g)})}\right]^{-} &= (\mathbf{A}_x^T\mathbf{A}_x \otimes \mathbf{A}_y^T\mathbf{A}_y \otimes \mathbf{A}_g^T\mathbf{W}_g\mathbf{A}_g)\mathbf{vec}(\mathbf{G}_{(g)}) \\
&+ (\mathbf{A}_x^T\mathbf{A}_x \otimes \mathbf{A}_y^T\mathbf{W}_y\mathbf{A}_y \otimes \mathbf{A}_g^T\mathbf{A}_g)\mathbf{vec}(\mathbf{G}_{(g)}) \\
&+ (\mathbf{A}_x^T\mathbf{W}_x\mathbf{A}_x \otimes \mathbf{A}_y^T\mathbf{A}_y \otimes \mathbf{A}_g^T\mathbf{A}_g)\mathbf{vec}(\mathbf{G}_{(g)})
\end{aligned} \tag{27}
$$

We can then update the core tensor with the same multiplicative update strategy as before:

$$
\left[\mathbf{vec}(\mathbf{G}_{(g)})\right]_i \longleftarrow \left[\mathbf{vec}(\mathbf{G}_{(g)})\right]_i \left(\frac{\left[\frac{\partial \mathcal{F}_1}{\partial \mathbf{vec}(\mathbf{G}_{(g)})}\right]_i^{-} + \lambda \left[\frac{\partial \mathcal{F}_2}{\partial \mathbf{vec}(\mathbf{G}_{(g)})}\right]_i^{-}}{\left[\frac{\partial \mathcal{F}_1}{\partial \mathbf{vec}(\mathbf{G}_{(g)})}\right]_i^{+} + \lambda \left[\frac{\partial \mathcal{F}_2}{\partial \mathbf{vec}(\mathbf{G}_{(g)})}\right]_i^{+}} \right) \tag{28}
$$

where i is the $i-$th entry in the vectorized core tensor $\mathbf{vec}(\mathbf{G}_{(g)})$.

S3 Factor matrix and core tensor normalization

To improve the interpretability of the output factor matrices and core tensor, an additional normalization step is performed at the end of every iteration. During this step, the columns of each factor matrix are modified such that all their columns sum to 1, and the core tensor is changed accordingly based on the transformations. This is accomplished using the following property:

$$
[\![\boldsymbol{\mathcal{G}}; \mathbf{A}_y, \mathbf{A}_x, \mathbf{A}_g]\!] = [\![\boldsymbol{\mathcal{G}} \times_1 \mathbf{U} \times_2 \mathbf{V} \times_3 \mathbf{W}; \mathbf{A}_y\mathbf{U}^{-1}, \mathbf{A}_x\mathbf{V}^{-1}, \mathbf{A}_g\mathbf{W}^{-1}]\!] \tag{29}
$$

Where $\mathbf{U} \in \mathbb{R}^{r_y \times r_y}, \mathbf{V} \in \mathbb{R}^{r_x \times r_x}$, and $\mathbf{W} \in \mathbb{R}^{r_g \times r_g}$ are diagonal matrices with entries equal to the sum of the column in their corresponding factor matrix, e.g. for all $i = j$, $[\mathbf{U}]_{i,j} = \sum_{k=1}^{r_y} [\mathbf{A}_y]_{k,j}$.

S4 Evaluation metrics

Three metrics were used to evaluate cross-validation performance in imputation experiments: mean absolute error (MAE), mean average percent error (MAPE), and coefficient of determination (R^2). Given a spot represented as a $n \times 1$ vector

of ground truth gene expressions $\mathbf{t}$ and vector of imputed gene expressions $\hat{\mathbf{t}}$ for that spot, each metric is calculated as follows:

$$\begin{aligned}
\text{MAE} &= \frac{1}{n}\left(\sum_{i=1}^{n}|\mathbf{t}_i - \hat{\mathbf{t}}_i|\right) \\
\text{MAPE} &= \frac{1}{n}\left(\sum_{i=1}^{n}|\frac{\mathbf{t}_i - \hat{\mathbf{t}}_i}{\mathbf{t}_i}|\right) \\
\text{R}^2 &= 1 - \frac{\sum\limits_{i=1}^{n}(\mathbf{t}_i - \hat{\mathbf{t}}_i)^2}{\sum\limits_{i=1}^{n}\left(\mathbf{t}_i - \frac{1}{n}\sum\limits_{j=1}^{n}\mathbf{t}_j\right)^2}
\end{aligned} \tag{30}$$

The Adjusted Rand Index (ARI) is calculated as:

$$\text{ARI} = \frac{\sum_{ij}\binom{n_{ij}}{2} - \left(\sum_i\binom{a_i}{2}\sum_i\binom{a_i}{2}\right)/\binom{n}{2}}{\frac{1}{2}\left(\sum_i\binom{a_i}{2} + \sum_i\binom{a_i}{2}\right) - \left(\sum_i\binom{a_i}{2}\sum_i\binom{a_i}{2}\right)/\binom{n}{2}}, \tag{31}$$

where n denotes the number of spots containing tissue, n_{ij} is the number of spots common between a cluster C_i and a tissue region R_i, $a_i = \sum_j n_{ij}$ is the number of spots common between C_i and all regions R_i, and b_j is the number of spots common between a region R_j and all clusters C_i.

S5 Imputation by spotwise cross-validation: extra results

We ran FIST an additional time on each of the three Visium datasets using rank= 200, as this was the suggested rank given in its original setting. We observed the results to be slightly worse when graph regularization is used compared to rank= 50, but we present them here in Table S1 for reference.

Table S1: Spotwise cross-validation results across three Visium datasets for FIST using rank= 200.

	**MBSA**			**MBSP**			**BRCA1**		
**Methods**	**MAE**	**MAPE**	**R2**	**MAE**	**MAPE**	**R2**	**MAE**	**MAPE**	**R2**
FIST rank=200, $\lambda = 0$	0.32	0.20	0.61	0.35	0.23	0.40	0.32	0.20	0.61
FIST rank=200, $\lambda = 0.1$	0.30	0.19	0.69	0.32	0.21	0.44	0.30	0.19	0.69

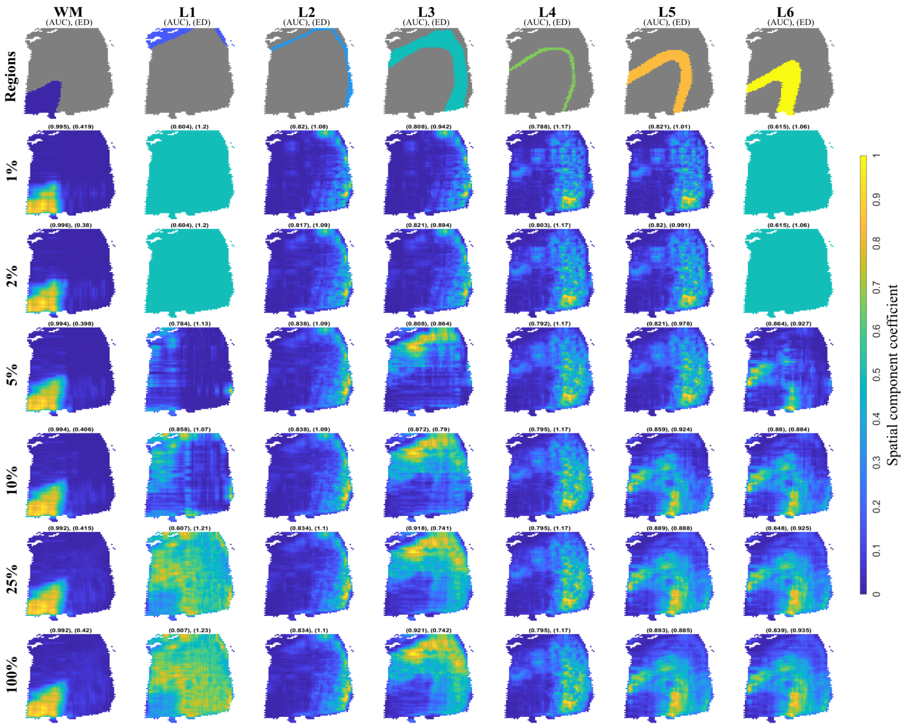

Figure S1: Matched spatial components for the seven human brain regions using increasing top percentages of the core tensor. Area Under the receiving operator Curve (AUC) and Euclidean Distance (ED) between the matched spatial component and region annotation is listed above each component.

Table S2: Table of p-values calculated from comparing gene components from GraphTucker with rank=$(30, 30, 7), \lambda = 1$ to sets of Human Brain layer-specific marker genes for regions L2-L6. WM and L1 regions are excluded due to having small number of marker genes. The p-values calculated from using the top 250, 500, 1000, 1500, and 2000 ranked genes in each region-matched spatial component's corresponding gene component. Num in top K refers to the number of marker genes that were present in top K rank of genes in the matched component. p-values were calculated using the hypergeometric test, using $N = 17891$ (total number of genes), $K \in \{250, 500, 1000, 1500, 2000\}$, and n = number of marker genes in the layer/region.

Region	Marker genes	Num in top 250	pval	Num in top 500	pval	Num in top 1000	pval	Num in top 1500	pval	Num in top 2000	pval
**L2**	50	5	6.49E-04	6	2.59E-03	11	7.86E-05	14	4.19E-05	18	3.66E-06
**L3**	51	4	5.56E-03	5	1.37E-02	8	6.93E-03	12	8.40E-04	14	1.07E-03
**L4**	23	2	4.06E-02	4	3.50E-03	4	3.67E-02	5	3.88E-02	5	1.06E-01
**L5**	59	5	1.39E-03	10	4.88E-06	12	8.50E-05	15	7.71E-05	18	4.90E-05
**L6**	67	4	1.44E-02	10	1.58E-05	18	1.68E-08	25	5.30E-11	26	4.26E-09

Table S3: Table of p-values calculated from comparing gene components from NMF with rank= 7 to sets of Human Brain layer-specific marker genes for regions L2-L6.

Region	Marker genes	Num in top 250	pval	Num in top 500	pval	Num in top 1000	pval	Num in top 1500	pval	Num in top 2000	pval
**L2**	50	6	6.66E-05	6	2.59E-03	12	1.47E-05	14	4.19E-05	20	1.44E-07
**L3**	51	5	7.12E-04	6	2.87E-03	11	9.52E-05	15	1.17E-05	16	8.75E-05
**L4**	23	2	4.06E-02	3	2.54E-02	4	3.67E-02	5	3.88E-02	5	1.06E-01
**L5**	59	9	1.20E-07	9	3.51E-05	12	8.50E-05	16	1.88E-05	19	1.29E-05
**L6**	67	3	6.73E-02	4	1.18E-01	7	7.95E-02	11	2.28E-02	13	3.27E-02

Table S4: Table of p-values calculated from comparing gene components from RNMTF with rank= $(50, 7), \gamma = 1, \rho = 0$ to sets of Human Brain layer-specific marker genes for regions L2-L6.

Region	Marker genes	Num in top 250	pval	Num in top 500	pval	Num in top 1000	pval	Num in top 1500	pval	Num in top 2000	pval
**L2**	50	0	1.00E+00	0	1.00E+00	0	1.00E+00	0	1.00E+00	1	9.97E-01
**L3**	51	0	1.00E+00	0	1.00E+00	0	1.00E+00	0	1.00E+00	0	1.00E+00
**L4**	23	3	3.88E-03	4	3.50E-03	4	3.67E-02	5	3.88E-02	8	2.51E-03
**L5**	59	6	1.70E-04	8	2.23E-04	11	3.72E-04	13	1.01E-03	17	1.73E-04
**L6**	67	0	1.00E+00	0	1.00E+00	3	7.31E-01	7	3.30E-01	10	2.11E-01

Table S5: Table of p-values calculated from comparing gene components from FIST with rank= 7, $\lambda = 1$ to sets of Human Brain layer-specific marker genes for regions L2-L6.

Region	Marker genes	Num in top 250	pval	Num in top 500	pval	Num in top 1000	pval	Num in top 1500	pval	Num in top 2000	pval
**L2**	50	4	5.18E-03	5	1.26E-02	9	1.62E-03	14	4.19E-05	17	1.63E-05
**L3**	51	2	1.59E-01	8	7.79E-05	9	1.87E-03	10	8.88E-03	15	3.20E-04
**L4**	23	1	2.77E-01	4	3.50E-03	4	3.67E-02	4	1.22E-01	5	1.06E-01
**L5**	59	5	1.39E-03	8	2.23E-04	13	1.76E-05	15	7.71E-05	17	1.73E-04
**L6**	67	4	1.44E-02	6	1.10E-02	13	7.29E-05	16	1.01E-04	18	2.96E-04

Table S6: Table of tested factor/component matrix ranks used for empirical runtime testing.

	**MBSA**	**MBSP**	**BRCA1**	**HB**	**MOSTA 9.5**	**MOSTA 11.5**
**NMF**	50	50	20	7	50	50
**RNMTF**	(50, 50)	(50, 50)	(20, 50)	(7, 50)	(50, 50)	(50, 50)
**FIST**	50	50	50	50	50	50
**GraphTucker**	(50, 50, 50)	(50, 50, 50)	(50, 50, 20)	(50, 50, 7)	(64, 64, 64)	(64, 64, 64)

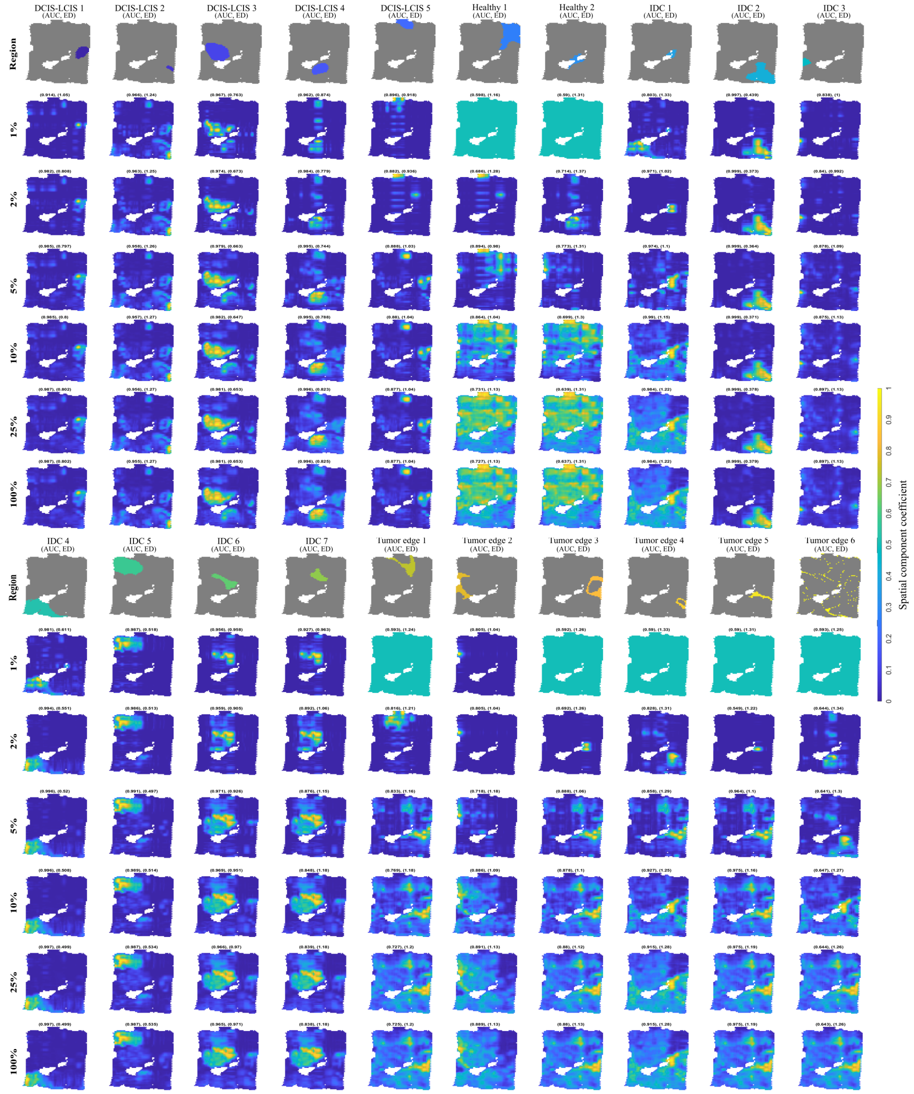

Figure S2: Matched spatial components for the twenty human breast cancer regions using increasing top percentages of the core tensor. Area Under the receiving operator Curve (AUC) and Euclidean Distance (ED) between the matched spatial component and region annotation is listed above each component.

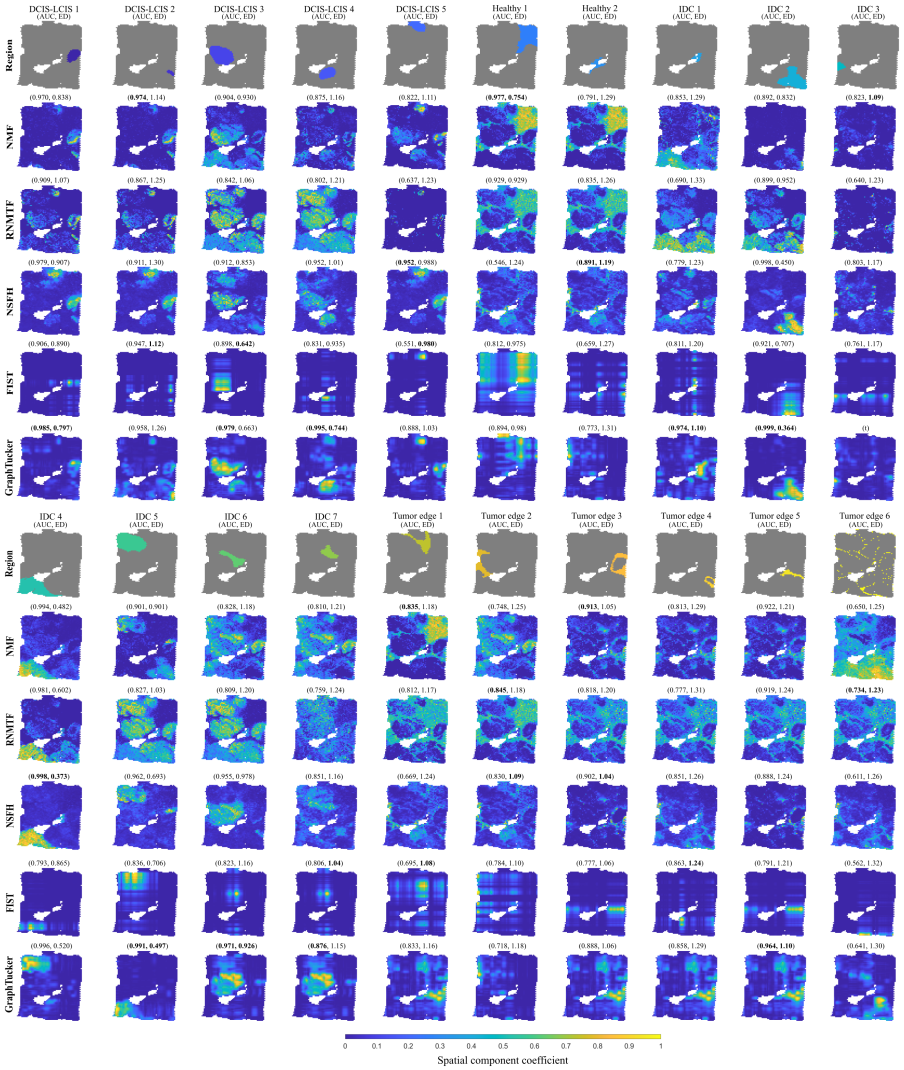

Figure S3: Complete results for comparison of all twenty annotated regions from the Visium human breast cancer dataset (BRCA1) with matched spatial components. Area Under the receiving operator Curve (AUC) and Euclidean Distance (ED) between the matched spatial component and region annotation is listed above each component. Best AUC and ED for each region are marked in bold. Components shown for GraphTucker are from using the top 5% of the core tensor.

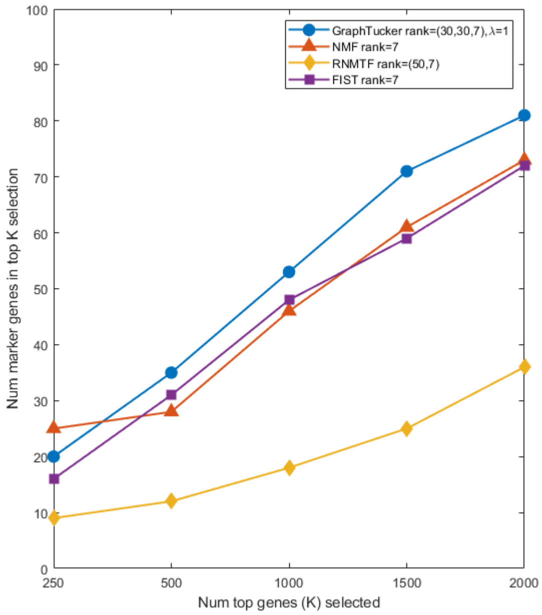

Figure S4: Plot showing the increasing number of genes present in the top $K \in \{250, 500, 1000, 1500, 2000\}$ ranked genes of GraphTucker and NMF region-matched gene components that overlap with corresponding layer-specific gene sets for human brain regions L2-L6. The total number of marker genes across these 5 region sets is 250.

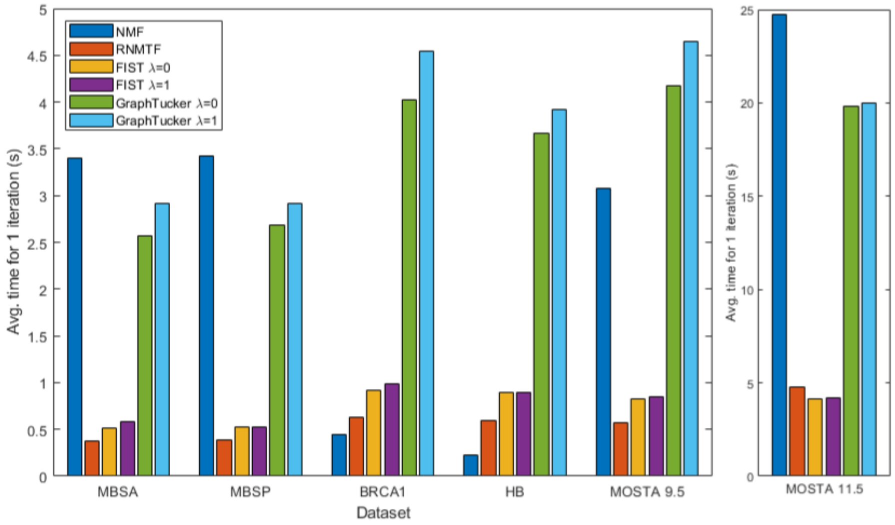

Figure S5: Run time analysis. The bar plots show the average run times for 1 iteration across the six datasets.
